# Predictive genetic plan for a captive population of the Chinese goral (*Naemorhedus griseus*) and prescriptive action for *ex situ* and *in situ* conservation management in Thailand

**DOI:** 10.1371/journal.pone.0234064

**Published:** 2020-06-04

**Authors:** Kornsuang Jangtarwan, Peerapong Kamsongkram, Navapong Subpayakom, Siwapech Sillapaprayoon, Narongrit Muangmai, Adisorn Kongphoemph, Apinya Wongsodchuen, Sanya Intapan, Wiyada Chamchumroon, Mongkol Safoowong, Surin Peyachoknagul, Prateep Duengkae, Kornsorn Srikulnath

**Affiliations:** 1 Laboratory of Animal Cytogenetics and Comparative Genomics (ACCG), Department of Genetics, Faculty of Science, Kasetsart University, Chatuchak, Bangkok, Thailand; 2 Special Research Unit for Wildlife Genomics (SRUWG), Department of Forest Biology, Faculty of Forestry, Kasetsart University, Chatuchak, Bangkok, Thailand; 3 Department of Fishery Biology, Faculty of Fisheries, Kasetsart University, Bangkok, Thailand; 4 Deparment of National Park, Wildlife and Plant Conservation, Ministry of Natural Resources and Environment, Bangkok, Thailand; 5 Center for Advanced Studies in Tropical Natural Resources, National Research University-Kasetsart University, Kasetsart University, Bangkok, Thailand (CASTNAR, NRU-KU, Thailand); 6 Center of Excellence on Agricultural Biotechnology (AG-BIO/PERDO-CHE), Bangkok, Thailand; 7 Amphibian Research Center, Hiroshima University, Kagamiyama, Higashihiroshima, Japan; Sichuan University, CHINA

## Abstract

Captive breeding programs for endangered species can increase population numbers for eventual reintroduction to the wild. Captive populations are typically small and isolated, which results in inbreeding and reduction of genetic variability, and may lead to an increased risk of extinction. The Omkoi Wildlife Breeding Center maintains the only Thai captive Chinese goral (*Naemorhedus griseus*) population, and has plans to reintroduce individuals into natural isolated populations. Genetic variability was assessed within the captive population using microsatellite data. Although no bottleneck was observed, genetic variability was low (allelic richness = 7.091 ± 0.756, *H*_e_ = 0.455 ± 0.219; *H*_e_ < *H*_o_) and 11 microsatellite loci were informative that likely reflect inbreeding. Estimates of small effective population size and limited numbers of founders, combined with wild-born individuals within subpopulations, tend to cause reduction of genetic variability over time in captive programs. This leads to low reproductive fitness and limited ability to adapt to environmental change, thereby increasing the risk of extinction. Management of captive populations as evolutionarily significant units with diverse genetic backgrounds offers an effective strategy for population recovery. Relocation of individuals among subpopulations, or introduction of newly captured wild individuals into the captive program will help to ensure the future security of Chinese goral. Implications for future conservation actions for the species are discussed herein.

## Introduction

We are currently facing a “biological annihilation” in terms of the diversity of fauna and flora as the Earth’s sixth mass extinction (or Anthropocene extinction) through the impacts of human activity [1]. Almost 50% of known animal species, including mammals, have been lost in recent decades. This suggests that the sixth mass extinction has already progressed further than was previously thought [1]. Native wildlife species must be preserved to maintain ecological balance. Active management of wildlife conservation strategies is necessary to ensure that ecosystems remain sustainable and intact. Tropical landscapes such as Thailand are globally important hotspots of biodiversity (e.g., Khonmee et al. [2]), although endemic species are being confronted by dramatic habitat loss as a result of climate change and anthropogenic activity [3]. The Chinese goral (*Naemorhedus griseus*) is a goat-like wild ungulate that is predominantly distributed across China, India, Myanmar, Thailand, and Vietnam [4–6]. In Thailand, the Chinese goral is only found in the northern region [7]. The general lack of education on wildlife means that hunters and the wider public do not recognize the biological and behavioral importance of Chinese gorals in the wild [8]. A previous national survey reported that excessive medicinal exploitation and habitat fragmentation have resulted in a dramatic and rapid decline in populations of Chinese goral throughout most of its range. The species now exists in small isolated populations with increased extinction risk [9]. Currently, 292 individuals have been identified in 11 areas distributed throughout the country (S1 Table). There is no dispersal between populations [8]. It was classified as Vulnerable by the International Union for Conservation of Nature (IUCN) in 2013, and also in a report in Appendix I of the Convention of International Trade on Endangered Species of Wild Fauna and Flora [10].

Conservation success requires an effective strategy for rapid action to be taken. Management of Chinese goral populations is important to guarantee their long-term survival in natural environments. To increase the Chinese goral population size, a captive breeding program is the only viable option because the species is almost extinct in the wild [11–13]. In 1993, a Chinese goral population was established outside their natural habitat (*ex situ*) at Omkoi Wildlife Breeding Center in Chiang Mai (17°28'14.8", 98°26'51.2"), Thailand. This program has been extremely successful and the population now comprises 73 individuals, with 67 captive-bred and six wild-born individuals captured or introduced between 2014 and 2019 ([14]; Adisorn Kongphoemph, personal communication). However, the existing captive breeding program has been conducted without genetic monitoring. Consequently, the risk of extinction may have increased as a result of inbreeding and low genetic variation due to the small founder population and currently small captive population [15]. We thus predict that these captive-bred Chinese gorals are less likely to survive when reintroduced to the wild. Research on the conservation genetics of this captive population is urgently required to increase their sustainability and chance of survival after reintroduction.

Maintenance of genetic diversity and demographic security are the primary goals for long-term conservation-population management. High genetic variation is the basis for adaptive evolution and survival and should be retained to promote the fitness of both individuals and the population [11–13]. By contrast, deleterious effects of inbreeding frequently occur in small captive populations, leading to decline of fitness and inbreeding depression [12,13]. Improvement in genetic variability can be achieved by minimizing the degree of relatedness (*r*) among individuals. This increases the likelihood of the re-establishment of a self-sustaining population [12,13]. Population size is also critical for practical conservation actions. High genetic variability with large effective population size (*N*_e_) encourages the success of future management options to ensure expansion of both wild and captive populations. In light of this scenario, the importance of genetic monitoring of the captive Chinese goral population was addressed by screening genetic variation using 11 microsatellite loci. Our aims were threefold: (i) establish a genetic baseline for the current captive population status, (ii) investigate the most important factors that determine the current population structure, and (iii) propose an *ex situ* genetic breeding management plan for recovery of wild populations by means of genetically cognizant restocking programs. These analyses have important implications for the selection of priority areas for the implementation of conservation management strategies to minimize the likelihood of population extinction.

## Materials and methods

### Specimen collection and DNA extraction

Seventy-three Chinese gorals were captured from the only captive breeding population in Thailand (Omkoi Wildlife Breeding Center). Human-mediated rotation of mating pairs is implemented in the captive population. However, the original sources of founders are unknown and wildlife staff did not track label information of mother/offspring from 1993. Blood samples were collected from the jugular vein using a 21-gauge needle attached to a 3 ml disposable syringe containing 10 mM ethylenediaminetetraacetic acid. All individuals were released immediately after blood collection. Total genomic DNA was extracted from the blood, following the standard salting-out protocol as described previously by Supikamolseni et al. [16], and used as the template for microsatellite genotyping. Detailed information on the sampled individuals is presented in S2 Table. The sex of each individual was identified by morphological observation. This research was conducted under the authority of the Department of National Parks, Wildlife and Plant Conservation (DNP) and the Ministry of Natural Resources and Environment, Thailand. Animal care and all experimental procedures were approved by the Animal Experiment Committee, DNP (approval no. TS.0909.704/2932, 2/7/2015 following the annual physical examination protocol) and conducted in accordance with the Regulations on Animal Experiments at Kasetsart University.

### Microsatellite genotyping

Eleven microsatellite primer sets were sourced from An et al. [17–18], having been developed originally from *Naemorhedus caudatus* (S3 Table). Cross-species amplification of microsatellite primers is frequently performed in conservation genetic and biodiversity research [19]. The 5′-end of the forward primer of each set of primers was labeled with fluorescent dye (6-FAM or HEX; Macrogen Inc., Seoul, Korea). PCR amplification was performed using 15 μl of 1× ThermoPol buffer containing 1.5 mM MgCl_2_, 0.2 mM dNTPs, 5.0 μM primers, 0.5 U *Taq* polymerase (Apsalagen Co. Ltd, Bangkok, Thailand), and 25 ng genomic DNA. The PCR protocol was as follows: initial denaturation at 94°C for 3 min, followed by 35 cycles of 94°C for 30 s, 50°C for 30 s, and 72°C for 1 min, with a final extension at 72°C for 7 min. The PCR products were detected by electrophoresis in 1% agarose gel. To decrease the influence of false alleles owing to the failure of PCR amplification, it was performed at least three times for each sample. A negative control (non-blood) specimen was prepared for each experiment. The absence of PCR products was also checked using 1% agarose gel electrophoresis after PCR. Fluorescent DNA fragment length analysis was subsequently performed using an ABI 3730XL automatic sequencer (Applied Biosystems, Foster City, CA, USA) at the DNA sequencing service of Macrogen Inc. Allelic size was determined using Peak Scanner version 1.0 software (Applied Biosystems). The genotypic da generated in this study were deposited in the Dryad Digital Repository. Dataset, https://doi.org/10.5061/dryad.wstqjq2hm.

### Microsatellite data analysis

We followed the same approaches used in a previous study of Asian woolly-necked storks (*Ciconia episcopus*) [12]. Allelic frequency, number of alleles (*A*), effective number of alleles (*N*_a_), observed heterozygosity (*H*_o_), expected heterozygosity (*H*_e_), and linkage equilibrium were calculated using Arlequin version 3.5.2.2 [20]. Given that the population was small, deviations from the Hardy-Weinberg equilibrium were evaluated at each locus using the Markov chain Monte Carlo (MCMC) approximation of Fisher’s exact test using the “genepop” function implemented in the package “*stats*” with R version 3.5.1 [21–23]. Welch’s *t*-test, which does not assume equal variance between samples, was used to test for significant differences between *H*_o_ and *H*_e_ using the “t.test” function in the package “*stats*” with R version 3.5.1 [23,24]. Allelic richness (*AR*) was calculated using FSTAT version 2.9.3 [25]. Micro-Checker version 2.2.3 was used to identify null allelic markers [26]. Polymorphic information content (PIC) was estimated using the Excel Microsatellite Toolkit [27] and calculated for each locus. Shannon's information index (*I*) and a fixation index (*F*) were calculated for each locus of the population using GenAlEx version 6.5 [28]. Effective population size (*N*_e_) was estimated as the number of breeding individuals that contributed to the population using the linkage disequilibrium method in NeEstimator version 2.01 [29].

To consider the possibility of sibling or parent-offspring pairs in the captive population, we determined whether the Chinese gorals were more related than random unrelated individuals. Relatedness values (*r*) were calculated for all pairs (comprising female-female, male-male, and male-female pairs), and mean pairwise *r* values based on allelic frequencies in the population were calculated at captivity using GenAlEx version 6.5 [28]. Individual and overall inbreeding coefficients (*F*_IS_) with 95% confidence intervals were calculated using the LynchRt estimator [30] as implemented in the program COANCESTRY [31]. Examination of *r* values and *F*_IS_ was conducted under the assumption that the averages did not differ significantly from random assortments of unrelated individuals. Parentage analysis and the probability that two individuals shared the same genotype were calculated using COLONY version 2.0.6.5 [32] and GIMLET version 1.3.3 [33], respectively. Mendelian inheritance was examined at every locus. Individuals sharing alleles from their putative parents at all loci were considered actual offspring of the pair. Cases in which pairing failed to match any of the two alleles of the putative parents at two or more loci were considered to be extra-pair paternity according to Chinese goral behavior [7,34].

The condition of heterozygosity abundance and changes in allelic frequency appropriations were examined in hereditarily bottlenecked populations using Bottleneck version 1.2.02 [35]. The Wilcoxon signed-rank test, with a two-phase mutation model (TPM) and stepwise mutation model (SMM), was used to obtain probabilities for excessive heterozygosity owing to the small sample sizes for loci and small sample size. The TPM was implemented with 95% single-step mutations and 5% multistep mutations, with variance among multiple steps set at 12 [36]. This test detects relatively short-term bottleneck events. To test for relatively long-term bottleneck events, the *M* ratio test [37] was performed using Arlequin version 3.5.2.2 [20]. The *M* ratio is the mean number of alleles in a population divided by the allelic size range and indicates reductions in both recent and historical population sizes. Principal component analysis (PCA) was performed to assess the overall relationship across individuals in the captive population using GenAlEx version 6.5. The model-based clustering method implemented in STRUCTURE version 2.3.3 was used to determine population structure [38]. Run length was set to 100,000 MCMC replicates after a burn-in period of 100,000 generations, using correlated allelic frequencies under a straight admixture model. Number of clusters (*K*) varied from 1 to 25, with 25 replicates for each value of *K*. The most probable number of bunches was dictated by plotting the log likelihood of the information (ln Pr (*X*|*K*)) [38] over the scope of tested K esteems before choosing the K esteem value at which ln Pr (*X*|*K*) settled. The ΔK strategy was applied utilizing Structure Harvester [39].

## Results

### Genetic variation of Chinese gorals in Omkoi Wildlife Breeding Center

All captive individuals were genotyped. Seventy-eight alleles were observed among all loci, with mean number of alleles per locus of 7.091 ± 0.756 (Table 1, S4 Table). Null alleles were frequently observed at all microsatellite loci, and all markers listed were similarly treated. All allelic frequencies showed significant departures from the Hardy-Weinberg equilibrium of the captive population, with multiple lines of evidence for linkage disequilibrium (S5 Table). Consequently, the population exhibited *F* values of 0.583. The PIC ranged from 0.103 to 0.769, and *I* ranged from 0.269 to 1.855 (Table 1, S4 Table). The *H*_o_ values ranged from 0.000 to 0.603 (mean ± SD: 0.191 ± 0.191) and the *H*_e_ values ranged from 0.106 to 0.800 (mean ± SD: 0.455 ± 0.219) (Table 1, S4 and S6 Tables). Welch’s *t*-test showed that *H*_o_ was significantly different from *H*_e_ in the population (*H*_o_ = 0.191 ± 0.191, *H*_e_ = 0.455 ± 0.219, *t* = −3.0183, df = 19.65, *p* < 0.05). The *AR* value of the population was 7.091 ± 0.756. Standard genetic diversity indices are summarized in Table 1 and S4 Table.

**Table 1:** Genetic diversity among 73 *Naemorhedus griseus* individuals based on 11 microsatellite loci.

Locality	Locus	*N*	*N*_a_	AR	*N*_e_	*I*	*H*_o_	*H*_e_	*M* ratio	PIC	*F*
Omkoi Wildlife Breeding Center	Mean	73	7.091	7.091	2.237	0.997	0.191	0.455	0.356	0.429	0.656
	S.D.	0	0.756	0.756	0.374	0.147	0.191	0.219	0.147	0.209	0.076

Sample size (*N*); number of alleles (*N*_a_); allelic richness (AR); number of effective alleles (*N*_e_); Shannon’s information index (*I*); observed heterozygosity (*H*_o_); expected heterozygosity (*H*_e_); *M* ratio test (*M* ratio); polymorphic information content (PIC); fixation index (*F*); *P*-value for comparison with the Hardy–Weinberg equilibrium (*P* < 0.05).

### Relatedness and estimation of population size in the captive population

A pairwise test was performed to determine the level of relatedness between individuals in the captive population. The mean pairwise *r* value of 2,628 goral pairs among the 73 sampled individuals was −0.011 ± 0.078. No Chinese goral pairs showed *r* < −0.25, there were 2,599 pairs with −0.25 < *r* < 0.25, and 29 pairs with 0.25 < *r* (S7 Table), which indicates that a proportion of the individuals in the population were closely related (*r* > 0.25). Mean *F*_IS_ was 0.041 ± 0.233, with individual *F*_IS_ ranging from −0.108 to 1.717 (Table 2, S8 Table).

**Table 2:** Inbreeding coefficients, relatedness, effective population size and ratio of effective population size and census population (*N*_e_*/N*) of *Naemorhedus griseus* at the Omkoi Wildlife Breeding Center.

Locality	*N*	*F*_IS_	Relatedness (*r*)	Estimated *N*_e_	95% CIs for *N*_e_	*N*_e_*/*N
Omkoi Wildlife Breeding Center	73	0.041±0.233	-0.011±0.078	49.6	36.3–69.5	0.679

Estimates were calculated using NeEstimator version 2.1, COANCESTRY, and GenAlEx version 6.5.

Detailed information for all *N*. *griseus* individuals is presented in S1 Table.

Sample size (*N*); inbreeding coefficient (*F*_IS_); effective population size (*N*_e_).

The *N*_e_ for individuals that contributed genetically to the current population was 49 (95% CI: 36.3–69.5) (Table 2). Simultaneously, parentage analysis of captive population individuals revealed that approximately one-third of all Chinese gorals as at least 21 of the total number originated from five breeding pairs (28.767%) (S9 Table). No genetic evidence for extra-pair paternity was observed. All paternities were assigned unequivocally. The combined likelihood of rejection for the microsatellites utilized was evaluated at 0.95. The probability of two Chinese gorals conveying an indistinguishable genotype was assessed at 6.93 × 10^−7^ (S10 Table).

### Population genetic structure

**Fig 1:**
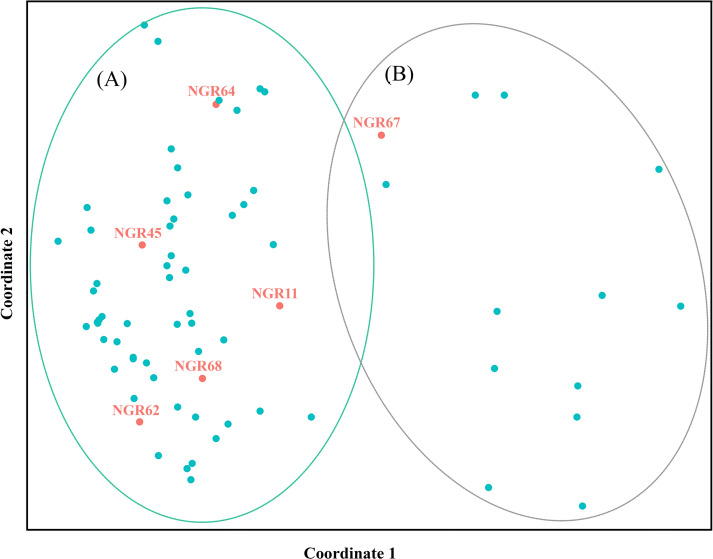
Principal component analysis of Chinese goral (*Naemorhedus griseus*) in Omkoi Wildlife Breeding Center, Thailand. Detailed information for all goral individuals is presented in S1 Table.

**Fig 2:**
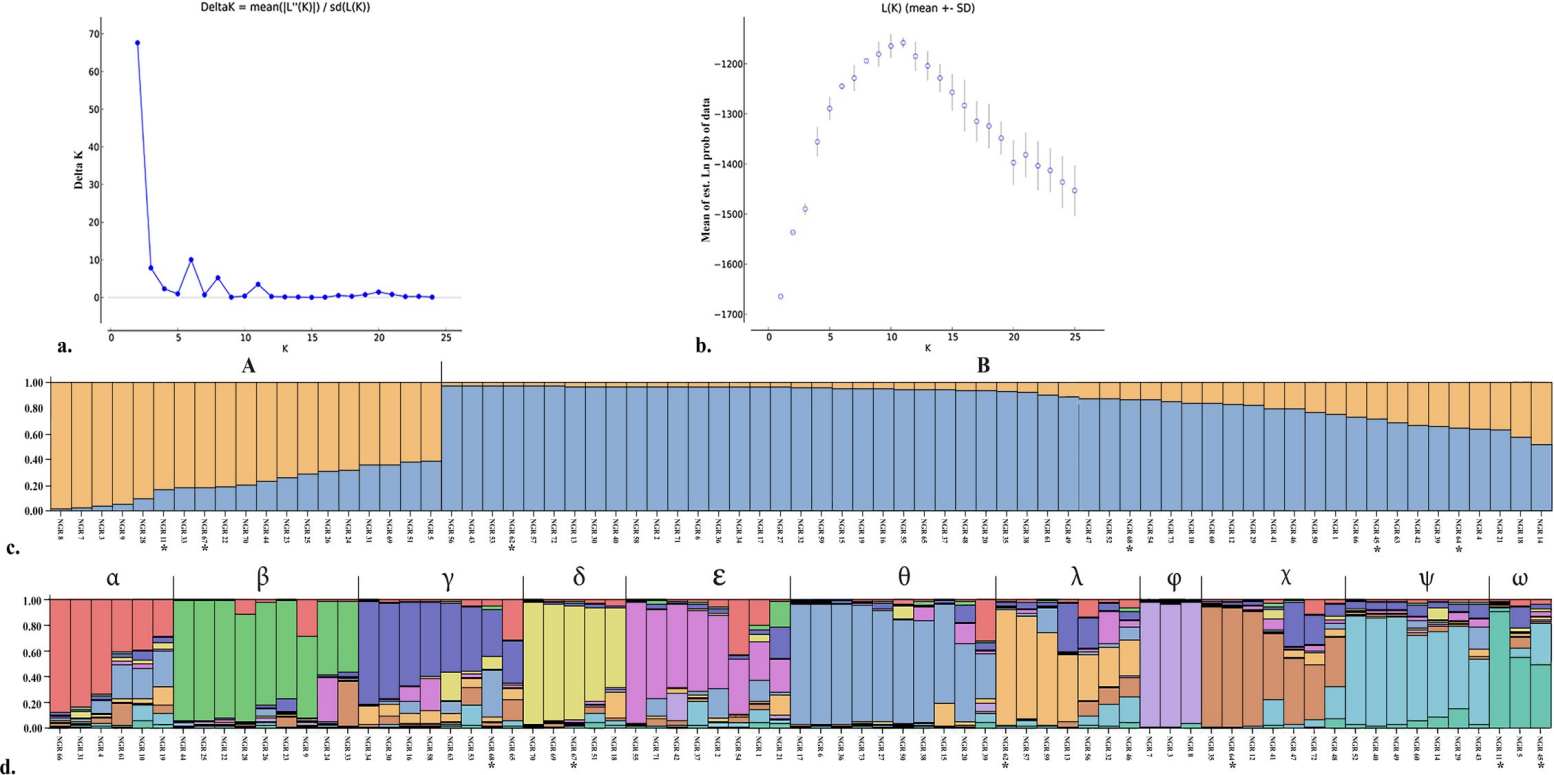
Population structure of 73 Chinese goral (*Naemorhedus griseus*) individuals. (a) Plot of Evanno’s Δ*K* and (b) plot of ln P (*K*). (c, d) Structure bar plots depicting the results of model-based clustering inferred for *K* = 2 (c) and *K* = 11 (d). Inferred genetic clusters are indicated by different colors. Each vertical bar on the *x*-axis represents an individual, and the *y*-axis represents the proportion of membership (posterior probability) in each genetic cluster. Chinese gorals are superimposed on the plot, with black vertical lines indicating the boundaries. Asterisks indicate wild-born individuals. Detailed information for all goral individuals is presented in S1 Table.

The Wilcoxon signed-rank tests for recent population bottlenecks gave SMM and TPM at 1.000 and 1.000, respectively in the captive population (normal L-shaped mode shift). The *M* ratio of population averaged 0.356 ± 0.147 (Table 1, S4 Table). The *M* ratio values were lower than the 0.68 threshold identified by Garza and Williamson [37], which indicates the presence of a historical reduction in population size. PCA revealed that the first, second, and third principal components accounted for 13.54%, 10.84%, and 8.40% of the total variation, respectively, and provided support for two tentatively differentiated Chinese goral groups (A and B) (Fig 1). Bayesian structural analysis revealed the highest posterior probability with one peak (*K* = 2) on the basis of Evanno’s Δ*K*, with all Chinese gorals grouped into two clusters (A and B) (Fig 2A and 2C). By contrast, Bayesian structural analysis based on the mean ln P (*K*) revealed one peak (*K* = 11) that provided evidence for 11 clusters (Fig 2B and 2D).

## Discussion

Human population growth has resulted in the continued decline of wildlife through the exploitation of natural resources and habitat encroachment [40], and many wildlife species are at risk of extinction, such as Chinese gorals in Korea [41]. Programs of reintroduction involving *ex situ* and *in situ* management of captive-bred individuals are necessary for recovery of Chinese goral populations in the wild, but policy decisions are required at the national level [42]. In captive programs, the primary goal is to develop a self-sustaining captive population by minimization of undesirable genetic changes owing to both selective pressure and selective neutrality (genetic drift) in the captive environment, avoidance of deleterious effects of inbreeding depression, and maintenance of a novel perspective on genetic management. Crucial parameters for estimation of genetic variability are *AR* and *H*_e_. Values of these parameters are 5–12 for *AR* and 0.6–0.9 for *H*_e_ in a genetically diverse captive mammalian population [43,44]. Allelic richness reflects the number of different alleles at any given locus in the population and is important for population size adaptation [45]. In the present study, the captive population exhibited low *AR* values, probably derived from the small initial founding population of eight individuals during the 2000s [46]. Heterozygosity is the proportion of heterozygous loci in a population and is important for immediate adaptation [47]. We observed that *H*_e_ was significantly higher than *H*_o_ in the captive population of Chinese gorals, which was suggestive of possible inbreeding owing to the small population size. This result was consistent with the positive *F*_IS_ value (average = 0.041) in the captive population. Reported values of *AR* and *H*_e_ in other deer populations include *AR* = 2.39–2.60 and *H*_e_ = 0.46–0.54 for Père David’s deer (*Elaphurus davidianus*) [48], and *AR* = 9.93–14.48 and *H*_e_ = 0.829–0.848 for Forest musk deer (*Moschus berezovskii*) [49]. These results suggest that the Chinese goral captive population may have grown by rapid proliferation of individuals with small *N*_e_ or *N*_e_/*N* (consensus population), similar to the reported population dynamics of the Asian Woollyneck (*Ciconia episcopus*) [12]. In the current study, we identified at least 49 individuals to be effective in transferring genetic components to the next generation, which suggests that the captive population is composed of related individuals. The polygynous behaviors of Chinese gorals are likely to cause a reduction in *N*_e_ for this species [50]. It is likely that one-third of all individuals originated from five breeding pairs in the captive population. However, sampling error might have occurred as a consequence of the small sample size [51]. Also, it could not be ruled out that null alleles were frequently observed at all microsatellite loci that occurred from microsatellite primers developed from different species [52]. This might result in substantial errors for data assessments of specific mating events to parentage exclusions and inbreeding [53]. Comparison of non-captive populations is necessary to examine whether these microsatellite markers are appropriate for use in this species or source population, while mitochondrial D-loop is an alternative approach to estimate empirical diversity and population [54]. Genetic monitoring of the captive population for the source of reintroduction is urgently required to address and plan our conservation program using all available microsatellites.

In contrast to *F*_IS_, the mean *r* value was almost zero in the captive population. However, the *F* estimations of the captive population were certain. These results were consistent with PCA and Bayesian structural analyses (*K* = 2) that resolved two tentative subpopulations in the captive population. The two genetic partitions may be a consequence of the different origins in the captive population derived from eight historically distinct founders in the wild and six wild-born individuals introduced into the program between 2014 and 2019 [46]. Unfortunately, the existing captive program was initiated without genetic background and provenance information for the founders. Group A within the captive population comprised two wild-born individuals and 17 captive-bred individuals. Thus, pairing between wild-born and several captive-bred individuals resulted in large numbers of the current captive individuals with low genetic variability. Interestingly, wild-born individuals in group A were caught in different locations: Huai Pu Ling (19°11'15.1", 98°06'15.8") and Doi Chiang Dao (19°23'59.0", 98°52'36.0"); nevertheless, the individuals exhibited a high degree of genetic similarity. This finding might also reflect the low degree of genetic variability in the different isolated populations, despite the disappearance of connecting groups owing to expansion of human settlements [55]. Group B comprised four wild-born individuals and 50 captive-bred individuals. Bottlenecks with low hereditary assorted variety frequently happen when few founders are taken from a declining wild population [56]. However, the present demographic analyses identified no recent bottlenecks in the captive population. This finding suggests that the captive population underwent recent expansion. An additional bottleneck might have occurred during reintroduction, when the captive population was genetically subdivided into several groups before release [57]. Careful examination of genetic variability with minimization of relatedness within the captive program is important to reduce inbreeding, and will allow more adaptive management decisions to be made. By prioritization of individuals with low relatedness for breeding pairs, population-level inbreeding and the loss of genetic variability can be mitigated by equalizing representation of individual genetic material within subpopulations as two groups or 11 groups (Fig 2). Here, we first recommend selection as a breeding pair of Chinese gorals from groups A and B or from between the 11 groups of subpopulations. Second, addition of newly captured wild individuals from isolated populations might be required frequently to expand the gene pool for new breeding programs, enhance genetic variability, and increase *N*_e_ or *N*_e_/*N* in the captive population to promote selection of release groups. When the opportunity arises for Chinese gorals to be reintroduced into the wild, the individuals will represent, as closely as possible, the genetic components of the original founders used to establish the captive population [58]. Further research into the genetic variation of wild isolated populations is needed to elucidate the underlying mechanisms that influence population dynamics, and to provide basic data for planning and implementation of effective management strategies to conserve Chinese gorals.

The genetic goals of the majority of captive breeding programs are based on maintenance of heterozygosity, but also relate to bottleneck effects in the pedigrees. Development of management plans to maintain genetic variability must consider the primary question, "How much genetic variability is required to retain long-term fitness and evolutionary potential in the captive population?” The finite availability of captive resources strictly limits the size and number of captive populations that can be managed, whereas loss of genetic variability is a function of time. For this reason, we need to propose a second question, “For how long must genetic variability be maintained?” The genetic diversity of a captive population is determined by the proportion of high heterozygosity over a specific period. Ideally, preservation of 90% heterozygosity of the founder will endure over a period of 100 years; however, it might also depend on generation time and sex behavior [59]. The current Chinese goral population consists of 73 individuals with *N*_e_ = 49. Multiple generations in the captive population are considered to reduce population size and increase the degree of inbreeding, as observed in other captive populations [60]. This suggests a low potential for recovery of the Chinese goral population in the wild. However, the date of a reintroduction event can be scheduled well in advance, and given that the Chinese goral has a predictable breeding pattern, males and females can be paired for the specific purpose of producing excess young for a particular reintroduction. Given that the captive program was begun using a small number of founders [61], introduction of large numbers of wild-caught Chinese goral into the captive population is required, although wild-caught individuals may be related or fail to breed, or their descendants may fail to reproduce. Alternatively, the current rapid development of biological technology, especially long-term storage by cryopreservation, will decrease dependence on populations of living Chinese gorals for preservation of gene pools. Cryopreservation of sperm and oocytes has been considered for conservation of Chinese gorals to preserve genetic variability under a limited effective population size [56]. However, cryopreservation is not always possible in conservation programs. We suggest that genetic variability is fully assessed before any recommendations are made for *ex situ* and *in situ* management. Thus, the present findings may help to streamline conservation efforts for Chinese gorals in Thailand.

**Fig 3:**
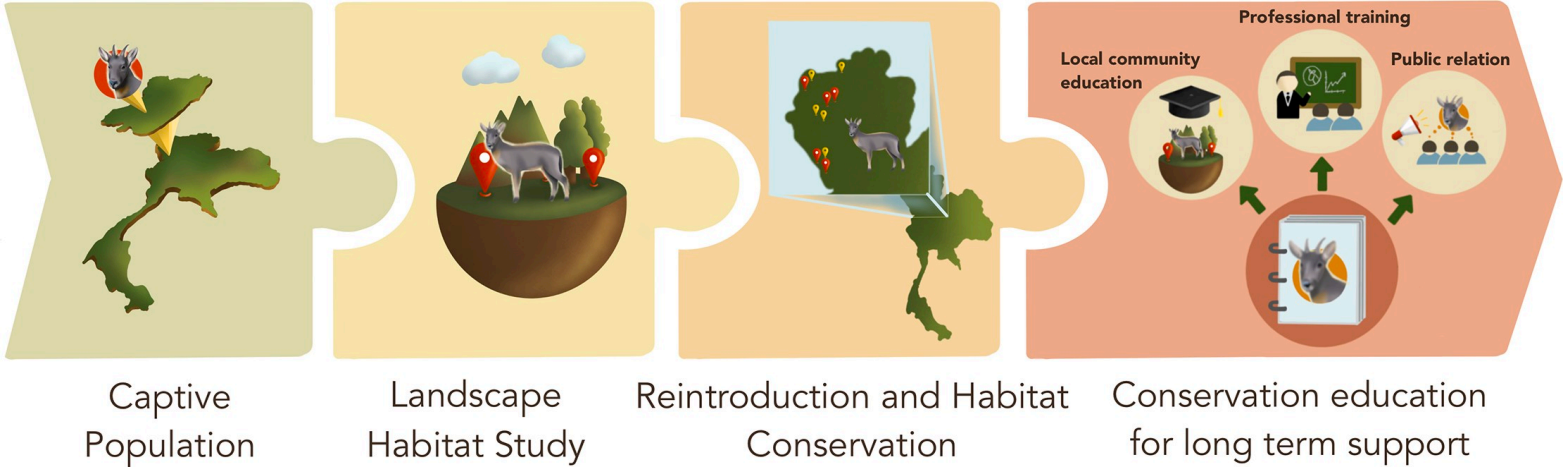
Conservation genetic roadmap for national Chinese goral (*Naemorhedus griseus*) conservation and recovery.

We propose a national ‘conservation genetic roadmap’ for Chinese goral conservation and recovery (Fig 3). This proposal entails the immediate implementation of several ‘no-regret’ measures that will act to slow or stop the decline of Chinese gorals. The process includes the maintenance of effective captive populations. Simultaneously, strategies for integrated genetic management and conservation, such as examination/monitoring of physical health, genetic monitoring, and assessment of the likelihood of behavioral anomalies, are required for future adaptation to environmental change in both captivity and in the wild [62–64]. Understanding the biology of Chinese gorals in their natural environment is difficult due to their complex behavior [8]. Investigation of appropriate landscape habitats as study sites is important for the survival of Chinese gorals. Administration and conservation of large herbivores are complex matters, principally because of their large spatial requirements. Intensive expansion of land transformation by humans is responsible for habitat fragmentation, and results in direct and indirect conflicts with wildlife over the remaining semi-natural habitats and resources [65]. Further in-depth field monitoring on the social structure and breeding behavior of Chinese gorals is needed to reveal the growth dynamics of isolated populations. Strengthening of future monitoring would be an effective means to survey the dynamics of genetic variability in isolated Chinese goral populations to maintain their long-term viability. Techniques such as non-invasive fecal sampling are required, followed by DNA extraction and analysis of population structure. On the basis of additional genetic monitoring, the translocation of individuals among geographically separated populations may improve the genetic variability of small isolated groups. The next important step for Chinese goral conservation is to re-establish a healthy population in their natural habitat (*in situ* conservation). Reintroduction of Chinese gorals into their natural environment should take place in habitat conservation and management areas. In addition, conservation education for long-term support is critical through professional training in the form of academic programs, workshops involving researchers and wildlife staff, internships, training courses, and fellowships. Determination of the most appropriate public relations and educational strategies using appropriate mass media (e.g., television, social media, radio, magazines, and newspapers) as well as local community education is needed. Collaboration with national governing sectors (DNP and/or National Research Council of Thailand, NRCT) will be necessary for documenting and monitoring the long-term effects of the proposed strategy on the diversity of Chinese gorals. Public-private partnerships and sustainable financing initiatives will also be launched with the aim of restoring, protecting, and creating novel Chinese goral habitats, as well as managing key threats and performing large-scale assessments of the conservation status of Chinese gorals to set priorities in relation to populations, protected areas, and other conservation challenges.

The present study on genetic monitoring of a captive Chinese goral population provides information useful to conservation management officers or wildlife staff for planning and monitoring of Chinese goral populations in the future. Standardized protocols used before, during, and after the reintroduction program can be adjusted and applied for the reintroduction of other species. Critically, the outcome of *ex situ* breeding programs may limit the ability of Chinese gorals to adapt to future environmental changes. It is clear that assessment of genetic variability is an important approach to maximize reproductive success and promote genetic variation in captive-bred individuals for subsequent release into the wild or to supplement captive populations. Long-term maintenance of populations requires implementation of a precise genetic breeding plan. Given the small sample size, the present results should be viewed with caution before implementation of a strict management or conservation strategy, to avoid genetic drift and ensure that a high proportion of the source variation is stabilized to minimize loss of genetic diversity. The present research project will contribute to the conservation of Chinese gorals in the wild for future generations. Such a ‘learning-by-doing’ approach ensures that these conservation strategies are robust to newly emerging pressures and threats. Implementation should be accompanied by research that examines impacts; the results can be used to modify and improve the implementation of effective measures.

## Supporting information

S1 Table Summary of Chinese goral (*Naemorhedus griseus*) in Thailand. (DOCX)

S2 Table Summary of Chinese goral (*Naemorhedus griseus*) individuals sampled. (DOCX)

S3 Table Microsatellite primers and sequences. (DOCX)

S4 Table Genetic diversity of 73 *Naemorhedus griseus* individuals based on 11 microsatellite loci. Detailed information for all *N*. *griseus* individuals is presented in S1 Table.
(DOCX)

S5 Table Pairwise differentiation of linkage disequilibrium of *Naemorhedus griseus* individuals in Omkoi Wildlife Breeding Center based on 11 microsatellite loci. Numbers indicate *p* values with 110 permutations.
(DOCX)

S6 Table Observed and expected heterozygosity of *Naemorhedus griseus* based on 11 microsatellite loci in Omkoi Wildlife Breeding Center and genetic bottlenecks for all individuals. Data were calculated using Bottleneck version 1.2.02 (Cornuet and Luikart, 1996). Detailed information for all *N*. *griseus* individuals is presented in S1 Table.
(DOCX)

S7 Table Pairwise genetic relatedness (*r*) for all 73 *Naemorhedus griseus* individuals. Detailed information for all *N*. *griseus* individuals is presented in S1 Table.
(DOCX)

S8 Table Pairwise inbreeding coefficients (*F*_IS_) for all 73 *Naemorhedus griseus* individuals. Detailed information for all *N*. *griseus* individuals is presented in S1 Table.
(DOCX)

S9 Table Parentage analysis of 73 *Naemorhedus griseus* individuals. Detailed information for all *N*. *griseus* individuals is presented in S1 Table.
(DOCX)

S10 Table Probability of identity estimated using Gimlet version 1.3.3 (Valière, 2002) of *Naemorhedus griseus* individuals based on 11 microsatellite loci. Detailed information for all *N*. *griseus* individuals is presented in S1 Table.
(DOCX)
