# Supplementary material for: Predictive genetic plan for a captive population of the Chinese goral (Naemorhedus griseus) and prescriptive action for ex situ and in situ conservation management in Thailand
Source: PLoS One. 2020 Jun 4;15(6):e0234064. doi: 10.1371/journal.pone.0234064 (PMC7272075; doi:10.1371/journal.pone.0234064)
Supplement: S1 Table — (DOCX) [file pone.0234064.s001.docx]

**Table S1.** Summary of Chinese goral (*Naemorhedus griseus*) in Thailand.

| **No.** | **Locality** | **longitude** | **latitude** |
| --- | --- | --- | --- |
| 1 | Doi Pha Hom Pok National Park | 19°59'47.8" | 99°08'39.6" |
| 2 | Doi Inthanon National Park | 18°32'32.7" | 98°33'00.5" |
| 3 | Nam Tok Mae Surin National Park Headquarters | 19°21'12.5" | 97°58'51.2" |
| 4 | Mae Ping National Park | 17°35'19.7" | 98°49'18.6" |
| 5 | Doi Chiang Dao | 19°23'59.0" | 98°52'36.0" |
| 6 | Mae Lao-Mae Sae Wildlife Sanctuary Office | 19°14'02.9" | 98°39'31.6" |
| 7 | Omkoi Wildlife Sanctuary | 17°28'41.1" | 98°27'27.6" |
| 8 | Lum Nam Pai Wildlife Sanctuary | 19°30'56.6" | 98°05'58.6" |
| 9 | San Pan Dan Wildlife Sanctuary | 19°33'23.5" | 98°07'10.4" |
| 10 | Mae Tuen Wildlife Sanctuary | 17°21'17.4" | 98°39'00.4" |
| 11 | Mae Chaem District | 18°47'41.7" | 98°20'59.5" |
