# Supplementary material for: Predictive genetic plan for a captive population of the Chinese goral (Naemorhedus griseus) and prescriptive action for ex situ and in situ conservation management in Thailand
Source: PLoS One. 2020 Jun 4;15(6):e0234064. doi: 10.1371/journal.pone.0234064 (PMC7272075; doi:10.1371/journal.pone.0234064)
Supplement: S2 Table — (DOCX) [file pone.0234064.s002.docx]

**Table S2.** Summary of Chinese goral (*Naemorhedus griseus*) individuals sampled.

| **No.** | **Code** | **Type** | **Sex** | **Microchip ID** | **Code ID** | **Locality** | **Locality**  **code** |  |
| --- | --- | --- | --- | --- | --- | --- | --- | --- |
| 1 | NGR01 | Goral | Female | 933.0764 | - | Omkoi Wildlife Breeding Center | OMK |  |
| 2 | NGR02 | Goral | Female | 933.0764 | - | Omkoi Wildlife Breeding Center | OMK |  |
| 3 | NGR03 | Goral | Male | 114973590A | - | Omkoi Wildlife Breeding Center | OMK |  |
| 4 | NGR04 | Goral | Male | 900.012 | - | Omkoi Wildlife Breeding Center | OMK |  |
| 5 | NGR05 | Goral | Female | 933.0764 | - | Omkoi Wildlife Breeding Center | OMK |  |
| 6 | NGR06 | Goral | Male | 982.0004 | - | Omkoi Wildlife Breeding Center | OMK |  |
| 7 | NGR07 | Goral | Female | 933.0764 | - | Omkoi Wildlife Breeding Center | OMK |  |
| 8 | NGR08 | Goral | Male | 900.012 | - | Omkoi Wildlife Breeding Center | OMK |  |
| 9 | NGR09 | Goral | Male | 982.0004 | - | Omkoi Wildlife Breeding Center | OMK |  |
| 10 | NGR10 | Goral | Male | 900.012 | - | Omkoi Wildlife Breeding Center | OMK |  |
| 11 | NGR11 | Goral | Female | - | - | Omkoi Wildlife Breeding Center | OMK |  |
| 12 | NGR12 | Goral | Male | - | OK-GR01 | Omkoi Wildlife Breeding Center | OMK |  |
| 13 | NGR13 | Goral | Male | - | OK-GR02 | Omkoi Wildlife Breeding Center | OMK |  |
| 14 | NGR14 | Goral | Male | - | OK-GR03 | Omkoi Wildlife Breeding Center | OMK |  |
| 15 | NGR15 | Goral | Male | - | OK-GR04 | Omkoi Wildlife Breeding Center | OMK |  |
| 16 | NGR16 | Goral | Male | - | OK-GR05 | Omkoi Wildlife Breeding Center | OMK |  |
| 17 | NGR17 | Goral | Male | - | OK-GR06 | Omkoi Wildlife Breeding Center | OMK |  |
| 18 | NGR18 | Goral | Male | - | OK-GR07 | Omkoi Wildlife Breeding Center | OMK |  |
| 19 | NGR19 | Goral | Female | - | OK-GR08 | Omkoi Wildlife Breeding Center | OMK |  |
| 20 | NGR20 | Goral | Male | - | OK-GR09 | Omkoi Wildlife Breeding Center | OMK |  |
| 21 | NGR21 | Goral | Male | - | OK-GR10 | Omkoi Wildlife Breeding Center | OMK |  |
| 22 | NGR22 | Goral | Male | - | OK-GR11 | Omkoi Wildlife Breeding Center | OMK |  |
| 23 | NGR23 | Goral | Male | - | OK-GR12 | Omkoi Wildlife Breeding Center | OMK |  |
| 24 | NGR24 | Goral | Male | - | OK-GR13 | Omkoi Wildlife Breeding Center | OMK |  |
| 25 | NGR25 | Goral | Female | - | OK-GR14 | Omkoi Wildlife Breeding Center | OMK |  |
| 26 | NGR26 | Goral | Female | - | OK-GR15 | Omkoi Wildlife Breeding Center | OMK |  |
| 27 | NGR27 | Goral | Female | - | OK-GR16 | Omkoi Wildlife Breeding Center | OMK |  |
| 28 | NGR28 | Goral | Male | - | OK-GR17 | Omkoi Wildlife Breeding Center | OMK |  |
| 29 | NGR29 | Goral | Female | - | OK-GR18 | Omkoi Wildlife Breeding Center | OMK |  |
| 30 | NGR30 | Goral | Female | - | OK-GR19 | Omkoi Wildlife Breeding Center | OMK |  |
| 31 | NGR31 | Goral | Female | - | OK-GR20 | Omkoi Wildlife Breeding Center | OMK |  |
| 32 | NGR32 | Goral | Female | - | OK-GR21 | Omkoi Wildlife Breeding Center | OMK |  |
| 33 | NGR33 | Goral | Female | - | OK-GR22 | Omkoi Wildlife Breeding Center | OMK |  |
| 34 | NGR34 | Goral | Male | - | OK-GR23 | Omkoi Wildlife Breeding Center | OMK |  |
| 35 | NGR35 | Goral | Male | - | OK-GR24 | Omkoi Wildlife Breeding Center | OMK |  |
| 36 | NGR36 | Goral | Female | - | OK-GR25 | Omkoi Wildlife Breeding Center | OMK |  |
| 37 | NGR37 | Goral | Male | - | OK-GR26 | Omkoi Wildlife Breeding Center | OMK |  |
| 38 | NGR38 | Goral | Female | - | OK-GR27 | Omkoi Wildlife Breeding Center | OMK |  |
| 39 | NGR39 | Goral | Female | - | OK-GR28 | Omkoi Wildlife Breeding Center | OMK |  |
| 40 | NGR40 | Goral | Female | - | OK-GR29 | Omkoi Wildlife Breeding Center | OMK |  |
| 41 | NGR41 | Goral | Male | - | OK-GR30 | Omkoi Wildlife Breeding Center | OMK |  |
| 42 | NGR42 | Goral | Male | - | OK-GR31 | Omkoi Wildlife Breeding Center | OMK |  |
| 43 | NGR43 | Goral | Female | - | OK-GR32 | Omkoi Wildlife Breeding Center | OMK |  |
| 44 | NGR44 | Goral | Female | - | OK-GR33 | Omkoi Wildlife Breeding Center | OMK |  |
| 45 | NGR45 | Goral | Female | - | OK-GR34 | Omkoi Wildlife Breeding Center | OMK |  |
| 46 | NGR46 | Goral | Male | - | OK-GR35 | Omkoi Wildlife Breeding Center | OMK |  |
| 47 | NGR47 | Goral | Female | - | OK-GR36 | Omkoi Wildlife Breeding Center | OMK |  |
| 48 | NGR48 | Goral | Female | - | OK-GR37 | Omkoi Wildlife Breeding Center | OMK |  |
| 49 | NGR49 | Goral | Male | - | OK-GR38 | Omkoi Wildlife Breeding Center | OMK |  |
| 50 | NGR50 | Goral | Male | - | OK-GR39 | Omkoi Wildlife Breeding Center | OMK |  |
| 51 | NGR51 | Goral | Female | - | OK-GR40 | Omkoi Wildlife Breeding Center | OMK |  |
| 52 | NGR52 | Goral | Female | - | OK-GR41 | Omkoi Wildlife Breeding Center | OMK |  |
| 53 | NGR53 | Goral | Female | - | OK-GR42 | Omkoi Wildlife Breeding Center | OMK |  |
| 54 | NGR54 | Goral | Female | - | OK-GR43 | Omkoi Wildlife Breeding Center | OMK |  |
| 55 | NGR55 | Goral | Male | - | OK-GR44 | Omkoi Wildlife Breeding Center | OMK |  |
| 56 | NGR56 | Goral | Female | - | OK-GR45 | Omkoi Wildlife Breeding Center | OMK |  |
| 57 | NGR57 | Goral | Male | - | OK-GR46 | Omkoi Wildlife Breeding Center | OMK |  |
| 58 | NGR58 | Goral | Female | - | OK-GR47 | Omkoi Wildlife Breeding Center | OMK |  |
| 59 | NGR59 | Goral | Female | - | OK-GR48 | Omkoi Wildlife Breeding Center | OMK |  |
| 60 | NGR60 | Goral | Male | - | OK-GR49 | Omkoi Wildlife Breeding Center | OMK |  |
| 61 | NGR61 | Goral | Male | - | OK-GR50 | Omkoi Wildlife Breeding Center | OMK |  |
| 62 | NGR62 | Goral | Female | - | OK-GR51 | Omkoi Wildlife Breeding Center | OMK |  |
| 63 | NGR63 | Goral | Female | - | OK-GR52 | Omkoi Wildlife Breeding Center | OMK |  |
| 64 | NGR64 | Goral | Male | - | OK-GR53 | Omkoi Wildlife Breeding Center | OMK |  |
| 65 | NGR65 | Goral | Female | - | OK-GR54 | Omkoi Wildlife Breeding Center | OMK |  |
| 66 | NGR66 | Goral | Male | - | OK-GR55 | Omkoi Wildlife Breeding Center | OMK |  |
| 67 | NGR67 | Goral | Female | - | OK-GR56 | Omkoi Wildlife Breeding Center | OMK |  |
| 68 | NGR68 | Goral | Female | - | OK-GR57 | Omkoi Wildlife Breeding Center | OMK |  |
| 69 | NGR69 | Goral | Male | - | OK-GR58 | Omkoi Wildlife Breeding Center | OMK |  |
| 70 | NGR70 | Goral | Female | - | OK-GR59 | Omkoi Wildlife Breeding Center | OMK |  |
| 71 | NGR71 | Goral | Female | - | OK-GR60 | Omkoi Wildlife Breeding Center | OMK |  |
| 72 | NGR72 | Goral | Female | - | OK-GR61 | Omkoi Wildlife Breeding Center | OMK |  |
| 73 | NGR73 | Goral | Male | - | OK-GR62 | Omkoi Wildlife Breeding Center | OMK |  |
| 74 | NGR74 | Goral | Female | - | OK-GR63 | Omkoi Wildlife Breeding Center | OMK |  |
| 75 | NGR75 | Goral | Female | - | OK-GR64 | Omkoi Wildlife Breeding Center | OMK |  |
