# Supplementary material for: Predictive genetic plan for a captive population of the Chinese goral (Naemorhedus griseus) and prescriptive action for ex situ and in situ conservation management in Thailand
Source: PLoS One. 2020 Jun 4;15(6):e0234064. doi: 10.1371/journal.pone.0234064 (PMC7272075; doi:10.1371/journal.pone.0234064)
Supplement: S3 Table — (DOCX) [file pone.0234064.s003.docx]

**Table S3.** Microsatellite primers and sequences.

| **Primer** | **Primer sequence 5′ to 3′** | **Reference** |
| --- | --- | --- |
| SY14 | GGAACCCTACCAATGCTCTG  CAAAGTGAATCGCCCGTC | An et al., 2010 |
| SY76 | AGGGTTTGCTTTTCAGGAC  CATCCATTACAGGAAGACTGC | An et al., 2010 |
| SY128 | TGACCCTTTGCTGTATCCTG  GGTGAGCCCAGAGAATCTTC | An et al., 2010 |
| SY129 | GAAAAAGAAGCACACACACG  AAGGTTTGTCCCCACATTC | An et al., 2010 |
| SY12B | TGACCCTTTGCTGTATCCTG  GGTGAGCCCAGAGAATCTTC | An et al., 2015 |
| SY84 | GAACTGAACTTGTTAGTATGTTGGG  TTGTTATGCTTGATGTTATTTTGTTAC | An et al., 2015 |
| SY84B | GGTCTGTGACATTAGTTCCTTTCC  GGCATTTTATTGGGGGAGAG | An et al., 2015 |
| SY93 | AATGAACAAAGAGGGTGTCAC  CATCCTTTTTCGTGGCTG | An et al., 2015 |
| SY259 | GCACCACAACAAAGAGGAGC  TGAAGACATAAGGGCGAACAG | An et al., 2015 |
| SY434 | AAGTGTCTGGGTTCTCTTTCTCTA  ATGTCAGTATGGGATGATGAATG | An et al., 2015 |
| SY449 | ATCTCCTGCCAAGTCCCC  TAGTAACGGCCGCCAGTG | An et al., 2015 |
