# Supplementary material for: Predictive genetic plan for a captive population of the Chinese goral (Naemorhedus griseus) and prescriptive action for ex situ and in situ conservation management in Thailand
Source: PLoS One. 2020 Jun 4;15(6):e0234064. doi: 10.1371/journal.pone.0234064 (PMC7272075; doi:10.1371/journal.pone.0234064)
Supplement: S4 Table — Detailed information for all N. griseus individuals is presented in S1 Table. (DOCX) [file pone.0234064.s004.docx]

**Table S4.** Genetic diversity of 73 *Naemorhedus griseus* individuals based on 11 microsatellite loci. Detailed information for all *N. griseus* individuals is presented in Table S1.

| **Pop** | **Locus** | ***N*** | ***Na*** | ***AR*** | ***Ne*** | ***I*** | ***Ho*** | ***He*** | ***M ratio*** | ***PIC*** | ***F*** | ***p-value*** |
| --- | --- | --- | --- | --- | --- | --- | --- | --- | --- | --- | --- | --- |
| **OMK** | **SY434F** | 73 | 7.000 | 7 | 1.499 | 0.737 | 0.082 | 0.335 | 0.318 | 0.315 | 0.753 | 0.000 |
|  | **SY14F** | 73 | 7.000 | 7 | 2.257 | 1.136 | 0.205 | 0.561 | 0.368 | 0.519 | 0.631 | 0.000 |
|  | **SY259F** | 73 | 4.000 | 4 | 1.118 | 0.269 | 0.000 | 0.106 | 0.571 | 0.103 | 1.000 | 0.000 |
|  | **SY12BF** | 73 | 11.000 | 11 | 4.867 | 1.855 | 0.356 | 0.800 | 0.379 | 0.769 | 0.552 | 0.000 |
|  | **SY93F** | 73 | 11.000 | 11 | 2.668 | 1.425 | 0.411 | 0.629 | 0.314 | 0.595 | 0.343 | 0.000 |
|  | **SY129F** | 73 | 7.000 | 7 | 1.500 | 0.744 | 0.068 | 0.335 | 0.28 | 0.315 | 0.794 | 0.000 |
|  | **SY76F** | 73 | 5.000 | 5 | 1.516 | 0.708 | 0.041 | 0.343 | 0.161 | 0.318 | 0.879 | 0.000 |
|  | **SY449F** | 73 | 4.000 | 4 | 1.348 | 0.549 | 0.055 | 0.260 | 0.571 | 0.245 | 0.788 | 0.000 |
|  | **SY128F** | 73 | 8.000 | 8 | 4.250 | 1.686 | 0.603 | 0.770 | 0.533 | 0.737 | 0.212 | 0.000 |
|  | **SY84BF** | 73 | 9.000 | 9 | 1.513 | 0.835 | 0.205 | 0.342 | 0.219 | 0.329 | 0.394 | 0.000 |
|  | **SY84F** | 73 | 5.000 | 5 | 2.068 | 1.024 | 0.068 | 0.520 | 0.2 | 0.479 | 0.867 | 0.000 |
|  | **Mean** | 73 | 7.091 | 7.091 | 2.237 | 0.997 | 0.191 | 0.455 | 0.356 | 0.429 | 0.656 |  |
|  | **SD** | 0 | 0.756 | 0.756 | 0.374 | 0.147 | 0.191 | 0.219 | 0.147 | 0.209 | 0.076 |  |
