# Supplementary material for: Predictive genetic plan for a captive population of the Chinese goral (Naemorhedus griseus) and prescriptive action for ex situ and in situ conservation management in Thailand
Source: PLoS One. 2020 Jun 4;15(6):e0234064. doi: 10.1371/journal.pone.0234064 (PMC7272075; doi:10.1371/journal.pone.0234064)
Supplement: S5 Table — Numbers indicate p values with 110 permutations. (DOCX) [file pone.0234064.s005.docx]

**Table S5.** Pairwise differentiation of linkage disequilibrium of *Naemorhedus griseus* individuals in Omkoi Wildlife Breeding Center based on 11 microsatellite loci. Numbers indicate *p*-values with 110 permutations.

| **Locus** | SY14 | SY76 | SY128 | SY129 | SY12B | SY84 | SY84B | SY93 | SY259 | SY434 | SY449 |
| --- | --- | --- | --- | --- | --- | --- | --- | --- | --- | --- | --- |
| SY14 | 0.00000 |  |  |  |  |  |  |  |  |  |  |
| SY76 | 0.00396 | 0.00000 |  |  |  |  |  |  |  |  |  |
| SY128 | 0.00099 | 0.00000 | 0.00000 |  |  |  |  |  |  |  |  |
| SY129 | 0.06614 | 0.09673 | 0.00000 | 0.00000 |  |  |  |  |  |  |  |
| SY12B | 0.15941 | 0.15525 | 0.12663 | 0.00446 | 0.00000 |  |  |  |  |  |  |
| SY84 | 0.36505 | 0.01386 | 0.00703 | 0.00970 | 0.65366 | 0.00000 |  |  |  |  |  |
| SY84B | 0.44426 | 0.08069 | 0.00119 | 0.00059 | 0.12218 | 0.00000 | 0.00000 |  |  |  |  |
| SY93 | 0.20406 | 0.00990 | 0.00208 | 0.02069 | 0.06446 | 0.00000 | 0.00000 | 0.00000 |  |  |  |
| SY259 | 0.00069 | 0.35673 | 0.14030 | 0.00030 | 0.11020 | 0.00030 | 0.02089 | 0.15970 | 0.00000 |  |  |
| SY434 | 0.00287 | 0.03812 | 0.00406 | 0.19416 | 0.05178 | 0.27901 | 0.55307 | 0.00990 | 0.00426 | 0.00000 |  |
| SY449 | 0.00000 | 0.02446 | 0.00000 | 0.00000 | 0.04901 | 0.00020 | 0.00495 | 0.04703 | 0.00000 | 0.00960 | 0.00000 |
