# Supplementary material for: Predictive genetic plan for a captive population of the Chinese goral (Naemorhedus griseus) and prescriptive action for ex situ and in situ conservation management in Thailand
Source: PLoS One. 2020 Jun 4;15(6):e0234064. doi: 10.1371/journal.pone.0234064 (PMC7272075; doi:10.1371/journal.pone.0234064)
Supplement: S6 Table — Data were calculated using Bottleneck version 1.2.02 (Cornuet and Luikart, 1996). Detailed information for all N. griseus individuals is presented in S1 Table. (DOCX) [file pone.0234064.s006.docx]

**Table S6.** Observed and expected heterozygosity of *Naemorhedus griseus* based on 11 microsatellite loci in Omkoi Wildlife Breeding Center and genetic bottlenecks for all individuals. Data were calculated using Bottleneck version 1.2.02 (Cornuet and Luikart, 1996). Detailed information for all N. griseus individuals is presented in Table S1.

| **Species** | **Captivity/wild** | ***H*_o_** | ***H*_e_** | ***p* value** | **Wilcoxon test** | | Mode-shift test | *M* ratio |
| --- | --- | --- | --- | --- | --- | --- | --- | --- |
|  |  |  |  |  | T.P.M. | S.M.M. |  |  |
| *Naemorhedus griseus* | Omkoi Wildlife Breeding Center | 0.191 ± 0.191 | 0.455 ± 0.219 | < 0.05 | 1.000 | 1.000 | normal L-shaped distribution | 0.356 ± 0.147 |
