# Supplementary material for: Predictive genetic plan for a captive population of the Chinese goral (Naemorhedus griseus) and prescriptive action for ex situ and in situ conservation management in Thailand
Source: PLoS One. 2020 Jun 4;15(6):e0234064. doi: 10.1371/journal.pone.0234064 (PMC7272075; doi:10.1371/journal.pone.0234064)
Supplement: S7 Table — Detailed information for all N. griseus individuals is presented in S1 Table. (DOCX) [file pone.0234064.s007.docx]

**Table S7.** Pairwise genetic relatedness (*r*) for all 73 *Naemorhedus griseus* individuals. Detailed information for all *N. griseus* individuals is presented in Table S1.

| **Sample 1** | **Sample 2** | ***r*** |
| --- | --- | --- |
| NGR1 | NGR2 | 0.033 |
| NGR1 | NGR3 | 0.036 |
| NGR2 | NGR3 | -0.121 |
| NGR1 | NGR4 | -0.012 |
| NGR2 | NGR4 | -0.051 |
| NGR3 | NGR4 | -0.063 |
| NGR1 | NGR5 | -0.025 |
| NGR2 | NGR5 | -0.032 |
| NGR3 | NGR5 | -0.136 |
| NGR4 | NGR5 | -0.068 |
| NGR1 | NGR6 | 0.013 |
| NGR2 | NGR6 | -0.075 |
| NGR3 | NGR6 | 0.426 |
| NGR4 | NGR6 | -0.077 |
| NGR5 | NGR6 | -0.100 |
| NGR1 | NGR7 | 0.013 |
| NGR2 | NGR7 | 0.044 |
| NGR3 | NGR7 | -0.121 |
| NGR4 | NGR7 | -0.051 |
| NGR5 | NGR7 | -0.050 |
| NGR6 | NGR7 | -0.049 |
| NGR1 | NGR8 | -0.088 |
| NGR2 | NGR8 | -0.146 |
| NGR3 | NGR8 | 0.276 |
| NGR4 | NGR8 | -0.117 |
| NGR5 | NGR8 | 0.399 |
| NGR6 | NGR8 | 1.159 |
| NGR7 | NGR8 | -0.146 |
| NGR1 | NGR9 | -0.032 |
| NGR2 | NGR9 | -0.035 |
| NGR3 | NGR9 | 0.290 |
| NGR4 | NGR9 | 0.035 |
| NGR5 | NGR9 | -0.090 |
| NGR6 | NGR9 | 0.196 |
| NGR7 | NGR9 | -0.056 |
| NGR8 | NGR9 | -0.164 |
| NGR1 | NGR10 | 0.037 |
| NGR2 | NGR10 | 0.055 |
| NGR3 | NGR10 | -0.121 |
| NGR4 | NGR10 | -0.064 |
| NGR5 | NGR10 | -0.013 |
| NGR6 | NGR10 | -0.057 |
| NGR7 | NGR10 | 0.035 |
| NGR8 | NGR10 | -0.146 |
| NGR9 | NGR10 | -0.045 |
| NGR1 | NGR11 | -0.062 |
| NGR2 | NGR11 | -0.044 |
| NGR3 | NGR11 | -0.139 |
| NGR4 | NGR11 | -0.069 |
| NGR5 | NGR11 | 0.454 |
| NGR6 | NGR11 | -0.111 |
| NGR7 | NGR11 | -0.026 |
| NGR8 | NGR11 | 0.127 |
| NGR9 | NGR11 | -0.101 |
| NGR10 | NGR11 | -0.052 |
| NGR1 | NGR12 | 0.022 |
| NGR2 | NGR12 | -0.038 |
| NGR3 | NGR12 | -0.037 |
| NGR4 | NGR12 | -0.010 |
| NGR5 | NGR12 | 0.019 |
| NGR6 | NGR12 | -0.100 |
| NGR7 | NGR12 | -0.057 |
| NGR8 | NGR12 | -0.146 |
| NGR9 | NGR12 | -0.090 |
| NGR10 | NGR12 | -0.020 |
| NGR11 | NGR12 | -0.075 |
| NGR1 | NGR13 | -0.046 |
| NGR2 | NGR13 | -0.029 |
| NGR3 | NGR13 | -0.026 |
| NGR4 | NGR13 | 0.058 |
| NGR5 | NGR13 | 0.004 |
| NGR6 | NGR13 | -0.119 |
| NGR7 | NGR13 | -0.011 |
| NGR8 | NGR13 | -0.146 |
| NGR9 | NGR13 | -0.127 |
| NGR10 | NGR13 | -0.011 |
| NGR11 | NGR13 | -0.052 |
| NGR12 | NGR13 | -0.002 |
| NGR1 | NGR14 | 0.017 |
| NGR2 | NGR14 | -0.006 |
| NGR3 | NGR14 | -0.037 |
| NGR4 | NGR14 | -0.068 |
| NGR5 | NGR14 | -0.053 |
| NGR6 | NGR14 | -0.092 |
| NGR7 | NGR14 | -0.025 |
| NGR8 | NGR14 | 0.218 |
| NGR9 | NGR14 | -0.064 |
| NGR10 | NGR14 | -0.014 |
| NGR11 | NGR14 | -0.043 |
| NGR12 | NGR14 | 0.046 |
| NGR13 | NGR14 | -0.051 |
| NGR1 | NGR15 | -0.006 |
| NGR2 | NGR15 | 0.026 |
| NGR3 | NGR15 | -0.121 |
| NGR4 | NGR15 | -0.069 |
| NGR5 | NGR15 | -0.069 |
| NGR6 | NGR15 | -0.049 |
| NGR7 | NGR15 | 0.114 |
| NGR8 | NGR15 | -0.146 |
| NGR9 | NGR15 | -0.074 |
| NGR10 | NGR15 | 0.016 |
| NGR11 | NGR15 | -0.044 |
| NGR12 | NGR15 | -0.075 |
| NGR13 | NGR15 | -0.029 |
| NGR14 | NGR15 | -0.043 |
| NGR1 | NGR16 | -0.064 |
| NGR2 | NGR16 | 0.039 |
| NGR3 | NGR16 | -0.073 |
| NGR4 | NGR16 | -0.019 |
| NGR5 | NGR16 | -0.014 |
| NGR6 | NGR16 | -0.119 |
| NGR7 | NGR16 | 0.042 |
| NGR8 | NGR16 | -0.146 |
| NGR9 | NGR16 | -0.146 |
| NGR10 | NGR16 | -0.029 |
| NGR11 | NGR16 | -0.070 |
| NGR12 | NGR16 | 0.001 |
| NGR13 | NGR16 | 0.073 |
| NGR14 | NGR16 | -0.069 |
| NGR15 | NGR16 | 0.024 |
| NGR1 | NGR17 | 0.013 |
| NGR2 | NGR17 | 0.032 |
| NGR3 | NGR17 | -0.121 |
| NGR4 | NGR17 | -0.057 |
| NGR5 | NGR17 | -0.050 |
| NGR6 | NGR17 | -0.049 |
| NGR7 | NGR17 | 0.120 |
| NGR8 | NGR17 | -0.146 |
| NGR9 | NGR17 | -0.033 |
| NGR10 | NGR17 | 0.023 |
| NGR11 | NGR17 | -0.038 |
| NGR12 | NGR17 | -0.057 |
| NGR13 | NGR17 | -0.023 |
| NGR14 | NGR17 | -0.037 |
| NGR15 | NGR17 | 0.102 |
| NGR16 | NGR17 | 0.030 |
| NGR1 | NGR18 | 0.007 |
| NGR2 | NGR18 | -0.017 |
| NGR3 | NGR18 | -0.046 |
| NGR4 | NGR18 | -0.010 |
| NGR5 | NGR18 | -0.063 |
| NGR6 | NGR18 | -0.093 |
| NGR7 | NGR18 | -0.017 |
| NGR8 | NGR18 | -0.146 |
| NGR9 | NGR18 | -0.015 |
| NGR10 | NGR18 | -0.024 |
| NGR11 | NGR18 | -0.026 |
| NGR12 | NGR18 | 0.036 |
| NGR13 | NGR18 | 0.007 |
| NGR14 | NGR18 | 0.068 |
| NGR15 | NGR18 | -0.035 |
| NGR16 | NGR18 | -0.061 |
| NGR17 | NGR18 | -0.028 |
| NGR1 | NGR19 | 0.045 |
| NGR2 | NGR19 | 0.004 |
| NGR3 | NGR19 | -0.079 |
| NGR4 | NGR19 | -0.001 |
| NGR5 | NGR19 | -0.043 |
| NGR6 | NGR19 | 0.003 |
| NGR7 | NGR19 | 0.048 |
| NGR8 | NGR19 | -0.146 |
| NGR9 | NGR19 | -0.039 |
| NGR10 | NGR19 | 0.040 |
| NGR11 | NGR19 | -0.026 |
| NGR12 | NGR19 | 0.003 |
| NGR13 | NGR19 | 0.028 |
| NGR14 | NGR19 | 0.036 |
| NGR15 | NGR19 | 0.029 |
| NGR16 | NGR19 | -0.040 |
| NGR17 | NGR19 | 0.036 |
| NGR18 | NGR19 | 0.059 |
| NGR1 | NGR20 | -0.006 |
| NGR2 | NGR20 | 0.100 |
| NGR3 | NGR20 | -0.121 |
| NGR4 | NGR20 | -0.057 |
| NGR5 | NGR20 | -0.069 |
| NGR6 | NGR20 | -0.049 |
| NGR7 | NGR20 | 0.056 |
| NGR8 | NGR20 | -0.146 |
| NGR9 | NGR20 | -0.052 |
| NGR10 | NGR20 | 0.004 |
| NGR11 | NGR20 | -0.056 |
| NGR12 | NGR20 | -0.075 |
| NGR13 | NGR20 | -0.041 |
| NGR14 | NGR20 | -0.055 |
| NGR15 | NGR20 | 0.038 |
| NGR16 | NGR20 | 0.200 |
| NGR17 | NGR20 | 0.073 |
| NGR18 | NGR20 | -0.047 |
| NGR19 | NGR20 | 0.017 |
| NGR1 | NGR21 | -0.051 |
| NGR2 | NGR21 | 0.065 |
| NGR3 | NGR21 | 0.120 |
| NGR4 | NGR21 | -0.069 |
| NGR5 | NGR21 | -0.069 |
| NGR6 | NGR21 | -0.015 |
| NGR7 | NGR21 | -0.022 |
| NGR8 | NGR21 | -0.086 |
| NGR9 | NGR21 | -0.101 |
| NGR10 | NGR21 | -0.029 |
| NGR11 | NGR21 | -0.063 |
| NGR12 | NGR21 | -0.076 |
| NGR13 | NGR21 | 0.047 |
| NGR14 | NGR21 | -0.043 |
| NGR15 | NGR21 | -0.040 |
| NGR16 | NGR21 | 0.215 |
| NGR17 | NGR21 | -0.034 |
| NGR18 | NGR21 | -0.044 |
| NGR19 | NGR21 | -0.024 |
| NGR20 | NGR21 | 0.182 |
| NGR1 | NGR22 | 0.018 |
| NGR2 | NGR22 | -0.091 |
| NGR3 | NGR22 | 0.014 |
| NGR4 | NGR22 | -0.047 |
| NGR5 | NGR22 | -0.114 |
| NGR6 | NGR22 | -0.081 |
| NGR7 | NGR22 | -0.001 |
| NGR8 | NGR22 | -0.107 |
| NGR9 | NGR22 | -0.111 |
| NGR10 | NGR22 | -0.098 |
| NGR11 | NGR22 | -0.095 |
| NGR12 | NGR22 | -0.099 |
| NGR13 | NGR22 | -0.051 |
| NGR14 | NGR22 | -0.095 |
| NGR15 | NGR22 | -0.010 |
| NGR16 | NGR22 | -0.011 |
| NGR17 | NGR22 | -0.001 |
| NGR18 | NGR22 | -0.095 |
| NGR19 | NGR22 | -0.084 |
| NGR20 | NGR22 | -0.065 |
| NGR21 | NGR22 | 0.078 |
| NGR1 | NGR23 | -0.100 |
| NGR2 | NGR23 | -0.107 |
| NGR3 | NGR23 | 0.081 |
| NGR4 | NGR23 | -0.086 |
| NGR5 | NGR23 | -0.050 |
| NGR6 | NGR23 | -0.164 |
| NGR7 | NGR23 | -0.063 |
| NGR8 | NGR23 | -0.164 |
| NGR9 | NGR23 | 0.236 |
| NGR10 | NGR23 | -0.089 |
| NGR11 | NGR23 | -0.111 |
| NGR12 | NGR23 | -0.035 |
| NGR13 | NGR23 | 0.013 |
| NGR14 | NGR23 | -0.111 |
| NGR15 | NGR23 | -0.082 |
| NGR16 | NGR23 | 0.008 |
| NGR17 | NGR23 | -0.018 |
| NGR18 | NGR23 | -0.043 |
| NGR19 | NGR23 | -0.100 |
| NGR20 | NGR23 | -0.009 |
| NGR21 | NGR23 | 0.061 |
| NGR22 | NGR23 | 0.083 |
| NGR1 | NGR24 | 0.209 |
| NGR2 | NGR24 | 0.137 |
| NGR3 | NGR24 | 0.009 |
| NGR4 | NGR24 | -0.071 |
| NGR5 | NGR24 | -0.087 |
| NGR6 | NGR24 | -0.052 |
| NGR7 | NGR24 | -0.019 |
| NGR8 | NGR24 | -0.136 |
| NGR9 | NGR24 | 0.090 |
| NGR10 | NGR24 | -0.014 |
| NGR11 | NGR24 | -0.096 |
| NGR12 | NGR24 | -0.094 |
| NGR13 | NGR24 | -0.078 |
| NGR14 | NGR24 | -0.077 |
| NGR15 | NGR24 | -0.038 |
| NGR16 | NGR24 | -0.010 |
| NGR17 | NGR24 | -0.025 |
| NGR18 | NGR24 | -0.048 |
| NGR19 | NGR24 | -0.023 |
| NGR20 | NGR24 | 0.070 |
| NGR21 | NGR24 | 0.134 |
| NGR22 | NGR24 | 0.075 |
| NGR23 | NGR24 | 0.200 |
| NGR1 | NGR25 | -0.030 |
| NGR2 | NGR25 | -0.070 |
| NGR3 | NGR25 | 0.000 |
| NGR4 | NGR25 | -0.040 |
| NGR5 | NGR25 | -0.105 |
| NGR6 | NGR25 | -0.110 |
| NGR7 | NGR25 | -0.025 |
| NGR8 | NGR25 | -0.136 |
| NGR9 | NGR25 | 0.126 |
| NGR10 | NGR25 | -0.077 |
| NGR11 | NGR25 | -0.074 |
| NGR12 | NGR25 | -0.069 |
| NGR13 | NGR25 | -0.077 |
| NGR14 | NGR25 | -0.074 |
| NGR15 | NGR25 | -0.044 |
| NGR16 | NGR25 | -0.048 |
| NGR17 | NGR25 | -0.031 |
| NGR18 | NGR25 | -0.006 |
| NGR19 | NGR25 | -0.062 |
| NGR20 | NGR25 | -0.022 |
| NGR21 | NGR25 | 0.051 |
| NGR22 | NGR25 | 0.517 |
| NGR23 | NGR25 | 0.334 |
| NGR24 | NGR25 | 0.202 |
| NGR1 | NGR26 | -0.033 |
| NGR2 | NGR26 | -0.002 |
| NGR3 | NGR26 | -0.148 |
| NGR4 | NGR26 | -0.096 |
| NGR5 | NGR26 | -0.096 |
| NGR6 | NGR26 | -0.086 |
| NGR7 | NGR26 | 0.041 |
| NGR8 | NGR26 | 0.381 |
| NGR9 | NGR26 | -0.056 |
| NGR10 | NGR26 | -0.011 |
| NGR11 | NGR26 | -0.053 |
| NGR12 | NGR26 | -0.103 |
| NGR13 | NGR26 | -0.057 |
| NGR14 | NGR26 | 0.120 |
| NGR15 | NGR26 | 0.022 |
| NGR16 | NGR26 | -0.049 |
| NGR17 | NGR26 | 0.029 |
| NGR18 | NGR26 | -0.053 |
| NGR19 | NGR26 | 0.002 |
| NGR20 | NGR26 | 0.038 |
| NGR21 | NGR26 | 0.072 |
| NGR22 | NGR26 | 0.075 |
| NGR23 | NGR26 | 0.085 |
| NGR24 | NGR26 | 0.115 |
| NGR25 | NGR26 | 0.123 |
| NGR1 | NGR27 | 0.013 |
| NGR2 | NGR27 | 0.044 |
| NGR3 | NGR27 | -0.121 |
| NGR4 | NGR27 | -0.051 |
| NGR5 | NGR27 | -0.050 |
| NGR6 | NGR27 | -0.049 |
| NGR7 | NGR27 | 0.087 |
| NGR8 | NGR27 | -0.146 |
| NGR9 | NGR27 | -0.056 |
| NGR10 | NGR27 | 0.035 |
| NGR11 | NGR27 | -0.026 |
| NGR12 | NGR27 | -0.057 |
| NGR13 | NGR27 | -0.011 |
| NGR14 | NGR27 | -0.025 |
| NGR15 | NGR27 | 0.068 |
| NGR16 | NGR27 | -0.003 |
| NGR17 | NGR27 | 0.075 |
| NGR18 | NGR27 | -0.017 |
| NGR19 | NGR27 | 0.048 |
| NGR20 | NGR27 | 0.084 |
| NGR21 | NGR27 | -0.022 |
| NGR22 | NGR27 | -0.046 |
| NGR23 | NGR27 | -0.036 |
| NGR24 | NGR27 | 0.008 |
| NGR25 | NGR27 | 0.002 |
| NGR26 | NGR27 | 0.068 |
| NGR1 | NGR28 | -0.053 |
| NGR2 | NGR28 | -0.045 |
| NGR3 | NGR28 | 0.033 |
| NGR4 | NGR28 | 0.106 |
| NGR5 | NGR28 | -0.127 |
| NGR6 | NGR28 | -0.095 |
| NGR7 | NGR28 | -0.028 |
| NGR8 | NGR28 | -0.164 |
| NGR9 | NGR28 | 0.452 |
| NGR10 | NGR28 | -0.054 |
| NGR11 | NGR28 | -0.074 |
| NGR12 | NGR28 | -0.111 |
| NGR13 | NGR28 | -0.050 |
| NGR14 | NGR28 | -0.074 |
| NGR15 | NGR28 | -0.047 |
| NGR16 | NGR28 | -0.118 |
| NGR17 | NGR28 | -0.011 |
| NGR18 | NGR28 | 0.019 |
| NGR19 | NGR28 | -0.005 |
| NGR20 | NGR28 | -0.003 |
| NGR21 | NGR28 | 0.029 |
| NGR22 | NGR28 | 0.026 |
| NGR23 | NGR28 | 0.400 |
| NGR24 | NGR28 | 0.242 |
| NGR25 | NGR28 | 0.296 |
| NGR26 | NGR28 | 0.120 |
| NGR27 | NGR28 | -0.001 |
| NGR1 | NGR29 | 0.152 |
| NGR2 | NGR29 | -0.007 |
| NGR3 | NGR29 | -0.121 |
| NGR4 | NGR29 | -0.024 |
| NGR5 | NGR29 | -0.041 |
| NGR6 | NGR29 | -0.097 |
| NGR7 | NGR29 | -0.007 |
| NGR8 | NGR29 | -0.009 |
| NGR9 | NGR29 | -0.105 |
| NGR10 | NGR29 | -0.002 |
| NGR11 | NGR29 | 0.224 |
| NGR12 | NGR29 | -0.005 |
| NGR13 | NGR29 | -0.020 |
| NGR14 | NGR29 | -0.028 |
| NGR15 | NGR29 | -0.016 |
| NGR16 | NGR29 | -0.017 |
| NGR17 | NGR29 | -0.019 |
| NGR18 | NGR29 | -0.030 |
| NGR19 | NGR29 | -0.009 |
| NGR20 | NGR29 | -0.037 |
| NGR21 | NGR29 | -0.035 |
| NGR22 | NGR29 | -0.014 |
| NGR23 | NGR29 | -0.077 |
| NGR24 | NGR29 | 0.110 |
| NGR25 | NGR29 | -0.031 |
| NGR26 | NGR29 | -0.053 |
| NGR27 | NGR29 | -0.007 |
| NGR28 | NGR29 | -0.096 |
| NGR1 | NGR30 | -0.046 |
| NGR2 | NGR30 | -0.029 |
| NGR3 | NGR30 | -0.121 |
| NGR4 | NGR30 | 0.002 |
| NGR5 | NGR30 | 0.004 |
| NGR6 | NGR30 | -0.119 |
| NGR7 | NGR30 | 0.015 |
| NGR8 | NGR30 | -0.146 |
| NGR9 | NGR30 | -0.127 |
| NGR10 | NGR30 | -0.011 |
| NGR11 | NGR30 | -0.052 |
| NGR12 | NGR30 | 0.041 |
| NGR13 | NGR30 | 0.044 |
| NGR14 | NGR30 | -0.051 |
| NGR15 | NGR30 | -0.003 |
| NGR16 | NGR30 | 0.073 |
| NGR17 | NGR30 | 0.003 |
| NGR18 | NGR30 | -0.042 |
| NGR19 | NGR30 | -0.022 |
| NGR20 | NGR30 | 0.012 |
| NGR21 | NGR30 | -0.048 |
| NGR22 | NGR30 | -0.051 |
| NGR23 | NGR30 | 0.040 |
| NGR24 | NGR30 | -0.050 |
| NGR25 | NGR30 | 0.019 |
| NGR26 | NGR30 | -0.003 |
| NGR27 | NGR30 | 0.042 |
| NGR28 | NGR30 | -0.072 |
| NGR29 | NGR30 | 0.023 |
| NGR1 | NGR31 | -0.014 |
| NGR2 | NGR31 | -0.001 |
| NGR3 | NGR31 | -0.130 |
| NGR4 | NGR31 | 0.132 |
| NGR5 | NGR31 | -0.082 |
| NGR6 | NGR31 | 0.346 |
| NGR7 | NGR31 | -0.002 |
| NGR8 | NGR31 | -0.009 |
| NGR9 | NGR31 | 0.167 |
| NGR10 | NGR31 | 0.003 |
| NGR11 | NGR31 | -0.057 |
| NGR12 | NGR31 | -0.066 |
| NGR13 | NGR31 | -0.011 |
| NGR14 | NGR31 | -0.047 |
| NGR15 | NGR31 | -0.002 |
| NGR16 | NGR31 | -0.079 |
| NGR17 | NGR31 | -0.014 |
| NGR18 | NGR31 | 0.015 |
| NGR19 | NGR31 | 0.030 |
| NGR20 | NGR31 | -0.033 |
| NGR21 | NGR31 | -0.076 |
| NGR22 | NGR31 | -0.117 |
| NGR23 | NGR31 | -0.062 |
| NGR24 | NGR31 | -0.028 |
| NGR25 | NGR31 | -0.037 |
| NGR26 | NGR31 | -0.039 |
| NGR27 | NGR31 | -0.002 |
| NGR28 | NGR31 | 0.201 |
| NGR29 | NGR31 | -0.045 |
| NGR30 | NGR31 | -0.061 |
| NGR1 | NGR32 | -0.005 |
| NGR2 | NGR32 | 0.001 |
| NGR3 | NGR32 | -0.026 |
| NGR4 | NGR32 | -0.072 |
| NGR5 | NGR32 | -0.023 |
| NGR6 | NGR32 | -0.075 |
| NGR7 | NGR32 | -0.018 |
| NGR8 | NGR32 | -0.146 |
| NGR9 | NGR32 | -0.028 |
| NGR10 | NGR32 | -0.007 |
| NGR11 | NGR32 | -0.059 |
| NGR12 | NGR32 | -0.029 |
| NGR13 | NGR32 | 0.051 |
| NGR14 | NGR32 | -0.021 |
| NGR15 | NGR32 | -0.027 |
| NGR16 | NGR32 | -0.015 |
| NGR17 | NGR32 | 0.028 |
| NGR18 | NGR32 | -0.031 |
| NGR19 | NGR32 | -0.011 |
| NGR20 | NGR32 | 0.009 |
| NGR21 | NGR32 | 0.058 |
| NGR22 | NGR32 | 0.025 |
| NGR23 | NGR32 | 0.040 |
| NGR24 | NGR32 | -0.067 |
| NGR25 | NGR32 | -0.072 |
| NGR26 | NGR32 | -0.064 |
| NGR27 | NGR32 | -0.018 |
| NGR28 | NGR32 | -0.049 |
| NGR29 | NGR32 | 0.038 |
| NGR30 | NGR32 | -0.044 |
| NGR31 | NGR32 | -0.053 |
| NGR1 | NGR33 | -0.025 |
| NGR2 | NGR33 | -0.050 |
| NGR3 | NGR33 | -0.106 |
| NGR4 | NGR33 | -0.093 |
| NGR5 | NGR33 | -0.085 |
| NGR6 | NGR33 | -0.090 |
| NGR7 | NGR33 | -0.008 |
| NGR8 | NGR33 | -0.164 |
| NGR9 | NGR33 | -0.078 |
| NGR10 | NGR33 | -0.047 |
| NGR11 | NGR33 | -0.090 |
| NGR12 | NGR33 | 0.325 |
| NGR13 | NGR33 | -0.069 |
| NGR14 | NGR33 | -0.066 |
| NGR15 | NGR33 | -0.017 |
| NGR16 | NGR33 | -0.062 |
| NGR17 | NGR33 | -0.008 |
| NGR18 | NGR33 | -0.048 |
| NGR19 | NGR33 | -0.020 |
| NGR20 | NGR33 | 0.001 |
| NGR21 | NGR33 | 0.024 |
| NGR22 | NGR33 | 0.100 |
| NGR23 | NGR33 | 0.087 |
| NGR24 | NGR33 | 0.081 |
| NGR25 | NGR33 | 0.078 |
| NGR26 | NGR33 | 0.132 |
| NGR27 | NGR33 | 0.020 |
| NGR28 | NGR33 | 0.086 |
| NGR29 | NGR33 | -0.036 |
| NGR30 | NGR33 | -0.016 |
| NGR31 | NGR33 | -0.061 |
| NGR32 | NGR33 | -0.029 |
| NGR1 | NGR34 | -0.046 |
| NGR2 | NGR34 | -0.029 |
| NGR3 | NGR34 | -0.026 |
| NGR4 | NGR34 | -0.041 |
| NGR5 | NGR34 | 0.004 |
| NGR6 | NGR34 | -0.119 |
| NGR7 | NGR34 | -0.011 |
| NGR8 | NGR34 | -0.146 |
| NGR9 | NGR34 | -0.127 |
| NGR10 | NGR34 | -0.011 |
| NGR11 | NGR34 | -0.052 |
| NGR12 | NGR34 | -0.002 |
| NGR13 | NGR34 | 0.139 |
| NGR14 | NGR34 | -0.051 |
| NGR15 | NGR34 | -0.029 |
| NGR16 | NGR34 | 0.073 |
| NGR17 | NGR34 | -0.023 |
| NGR18 | NGR34 | -0.042 |
| NGR19 | NGR34 | -0.022 |
| NGR20 | NGR34 | 0.013 |
| NGR21 | NGR34 | 0.047 |
| NGR22 | NGR34 | -0.051 |
| NGR23 | NGR34 | 0.068 |
| NGR24 | NGR34 | -0.023 |
| NGR25 | NGR34 | -0.023 |
| NGR26 | NGR34 | -0.002 |
| NGR27 | NGR34 | 0.044 |
| NGR28 | NGR34 | -0.045 |
| NGR29 | NGR34 | -0.020 |
| NGR30 | NGR34 | 0.098 |
| NGR31 | NGR34 | -0.061 |
| NGR32 | NGR34 | 0.051 |
| NGR33 | NGR34 | -0.015 |
| NGR1 | NGR35 | 0.003 |
| NGR2 | NGR35 | -0.057 |
| NGR3 | NGR35 | -0.037 |
| NGR4 | NGR35 | 0.040 |
| NGR5 | NGR35 | 0.000 |
| NGR6 | NGR35 | -0.110 |
| NGR7 | NGR35 | -0.057 |
| NGR8 | NGR35 | -0.146 |
| NGR9 | NGR35 | -0.063 |
| NGR10 | NGR35 | -0.038 |
| NGR11 | NGR35 | -0.075 |
| NGR12 | NGR35 | 0.143 |
| NGR13 | NGR35 | 0.047 |
| NGR14 | NGR35 | 0.028 |
| NGR15 | NGR35 | -0.075 |
| NGR16 | NGR35 | 0.001 |
| NGR17 | NGR35 | -0.012 |
| NGR18 | NGR35 | 0.052 |
| NGR19 | NGR35 | 0.019 |
| NGR20 | NGR35 | -0.030 |
| NGR21 | NGR35 | -0.085 |
| NGR22 | NGR35 | -0.099 |
| NGR23 | NGR35 | 0.055 |
| NGR24 | NGR35 | -0.103 |
| NGR25 | NGR35 | -0.069 |
| NGR26 | NGR35 | -0.103 |
| NGR27 | NGR35 | -0.057 |
| NGR28 | NGR35 | -0.041 |
| NGR29 | NGR35 | -0.014 |
| NGR30 | NGR35 | 0.041 |
| NGR31 | NGR35 | -0.051 |
| NGR32 | NGR35 | 0.043 |
| NGR33 | NGR35 | -0.039 |
| NGR34 | NGR35 | -0.002 |
| NGR1 | NGR36 | 0.013 |
| NGR2 | NGR36 | 0.044 |
| NGR3 | NGR36 | -0.121 |
| NGR4 | NGR36 | -0.051 |
| NGR5 | NGR36 | -0.050 |
| NGR6 | NGR36 | -0.049 |
| NGR7 | NGR36 | 0.132 |
| NGR8 | NGR36 | -0.146 |
| NGR9 | NGR36 | -0.056 |
| NGR10 | NGR36 | 0.035 |
| NGR11 | NGR36 | -0.026 |
| NGR12 | NGR36 | -0.057 |
| NGR13 | NGR36 | -0.011 |
| NGR14 | NGR36 | -0.025 |
| NGR15 | NGR36 | 0.114 |
| NGR16 | NGR36 | 0.042 |
| NGR17 | NGR36 | 0.120 |
| NGR18 | NGR36 | -0.017 |
| NGR19 | NGR36 | 0.048 |
| NGR20 | NGR36 | 0.056 |
| NGR21 | NGR36 | -0.022 |
| NGR22 | NGR36 | -0.001 |
| NGR23 | NGR36 | -0.063 |
| NGR24 | NGR36 | -0.019 |
| NGR25 | NGR36 | -0.025 |
| NGR26 | NGR36 | 0.041 |
| NGR27 | NGR36 | 0.087 |
| NGR28 | NGR36 | -0.028 |
| NGR29 | NGR36 | -0.007 |
| NGR30 | NGR36 | 0.015 |
| NGR31 | NGR36 | -0.002 |
| NGR32 | NGR36 | -0.018 |
| NGR33 | NGR36 | -0.008 |
| NGR34 | NGR36 | -0.011 |
| NGR35 | NGR36 | -0.057 |
| NGR1 | NGR37 | 0.119 |
| NGR2 | NGR37 | 0.010 |
| NGR3 | NGR37 | -0.006 |
| NGR4 | NGR37 | 0.038 |
| NGR5 | NGR37 | -0.013 |
| NGR6 | NGR37 | -0.017 |
| NGR7 | NGR37 | -0.009 |
| NGR8 | NGR37 | -0.088 |
| NGR9 | NGR37 | -0.064 |
| NGR10 | NGR37 | 0.002 |
| NGR11 | NGR37 | -0.049 |
| NGR12 | NGR37 | 0.023 |
| NGR13 | NGR37 | -0.034 |
| NGR14 | NGR37 | -0.011 |
| NGR15 | NGR37 | -0.027 |
| NGR16 | NGR37 | -0.032 |
| NGR17 | NGR37 | -0.009 |
| NGR18 | NGR37 | -0.022 |
| NGR19 | NGR37 | -0.001 |
| NGR20 | NGR37 | -0.027 |
| NGR21 | NGR37 | -0.027 |
| NGR22 | NGR37 | 0.064 |
| NGR23 | NGR37 | -0.068 |
| NGR24 | NGR37 | -0.001 |
| NGR25 | NGR37 | 0.037 |
| NGR26 | NGR37 | -0.054 |
| NGR27 | NGR37 | -0.009 |
| NGR28 | NGR37 | -0.086 |
| NGR29 | NGR37 | 0.030 |
| NGR30 | NGR37 | 0.008 |
| NGR31 | NGR37 | -0.053 |
| NGR32 | NGR37 | 0.019 |
| NGR33 | NGR37 | -0.079 |
| NGR34 | NGR37 | -0.034 |
| NGR35 | NGR37 | 0.004 |
| NGR36 | NGR37 | -0.009 |
| NGR1 | NGR38 | 0.127 |
| NGR2 | NGR38 | 0.020 |
| NGR3 | NGR38 | -0.006 |
| NGR4 | NGR38 | -0.005 |
| NGR5 | NGR38 | -0.050 |
| NGR6 | NGR38 | 0.008 |
| NGR7 | NGR38 | 0.108 |
| NGR8 | NGR38 | -0.088 |
| NGR9 | NGR38 | -0.056 |
| NGR10 | NGR38 | 0.011 |
| NGR11 | NGR38 | -0.049 |
| NGR12 | NGR38 | -0.057 |
| NGR13 | NGR38 | -0.034 |
| NGR14 | NGR38 | -0.048 |
| NGR15 | NGR38 | 0.090 |
| NGR16 | NGR38 | 0.018 |
| NGR17 | NGR38 | 0.108 |
| NGR18 | NGR38 | -0.040 |
| NGR19 | NGR38 | 0.024 |
| NGR20 | NGR38 | 0.044 |
| NGR21 | NGR38 | -0.046 |
| NGR22 | NGR38 | 0.114 |
| NGR23 | NGR38 | -0.063 |
| NGR24 | NGR38 | 0.026 |
| NGR25 | NGR38 | 0.020 |
| NGR26 | NGR38 | 0.017 |
| NGR27 | NGR38 | 0.063 |
| NGR28 | NGR38 | -0.040 |
| NGR29 | NGR38 | -0.031 |
| NGR30 | NGR38 | -0.009 |
| NGR31 | NGR38 | -0.026 |
| NGR32 | NGR38 | -0.018 |
| NGR33 | NGR38 | -0.008 |
| NGR34 | NGR38 | -0.034 |
| NGR35 | NGR38 | -0.057 |
| NGR36 | NGR38 | 0.108 |
| NGR37 | NGR38 | 0.106 |
| NGR1 | NGR39 | 0.013 |
| NGR2 | NGR39 | 0.044 |
| NGR3 | NGR39 | 0.243 |
| NGR4 | NGR39 | -0.069 |
| NGR5 | NGR39 | -0.050 |
| NGR6 | NGR39 | 0.096 |
| NGR7 | NGR39 | 0.042 |
| NGR8 | NGR39 | -0.009 |
| NGR9 | NGR39 | -0.056 |
| NGR10 | NGR39 | 0.035 |
| NGR11 | NGR39 | -0.044 |
| NGR12 | NGR39 | -0.057 |
| NGR13 | NGR39 | -0.029 |
| NGR14 | NGR39 | -0.025 |
| NGR15 | NGR39 | 0.042 |
| NGR16 | NGR39 | -0.048 |
| NGR17 | NGR39 | 0.030 |
| NGR18 | NGR39 | -0.026 |
| NGR19 | NGR39 | 0.038 |
| NGR20 | NGR39 | 0.012 |
| NGR21 | NGR39 | -0.031 |
| NGR22 | NGR39 | -0.082 |
| NGR23 | NGR39 | -0.107 |
| NGR24 | NGR39 | -0.028 |
| NGR25 | NGR39 | -0.070 |
| NGR26 | NGR39 | -0.004 |
| NGR27 | NGR39 | 0.042 |
| NGR28 | NGR39 | -0.047 |
| NGR29 | NGR39 | -0.007 |
| NGR30 | NGR39 | -0.029 |
| NGR31 | NGR39 | 0.143 |
| NGR32 | NGR39 | -0.009 |
| NGR33 | NGR39 | -0.043 |
| NGR34 | NGR39 | -0.029 |
| NGR35 | NGR39 | -0.066 |
| NGR36 | NGR39 | 0.042 |
| NGR37 | NGR39 | -0.009 |
| NGR38 | NGR39 | 0.018 |
| NGR1 | NGR40 | -0.005 |
| NGR2 | NGR40 | 0.024 |
| NGR3 | NGR40 | -0.121 |
| NGR4 | NGR40 | -0.017 |
| NGR5 | NGR40 | -0.023 |
| NGR6 | NGR40 | -0.075 |
| NGR7 | NGR40 | 0.006 |
| NGR8 | NGR40 | -0.146 |
| NGR9 | NGR40 | -0.074 |
| NGR10 | NGR40 | 0.017 |
| NGR11 | NGR40 | -0.035 |
| NGR12 | NGR40 | 0.013 |
| NGR13 | NGR40 | -0.020 |
| NGR14 | NGR40 | 0.003 |
| NGR15 | NGR40 | -0.003 |
| NGR16 | NGR40 | -0.017 |
| NGR17 | NGR40 | -0.006 |
| NGR18 | NGR40 | -0.007 |
| NGR19 | NGR40 | 0.013 |
| NGR20 | NGR40 | -0.024 |
| NGR21 | NGR40 | -0.013 |
| NGR22 | NGR40 | -0.001 |
| NGR23 | NGR40 | -0.077 |
| NGR24 | NGR40 | -0.055 |
| NGR25 | NGR40 | -0.018 |
| NGR26 | NGR40 | -0.040 |
| NGR27 | NGR40 | 0.006 |
| NGR28 | NGR40 | -0.083 |
| NGR29 | NGR40 | 0.104 |
| NGR30 | NGR40 | 0.023 |
| NGR31 | NGR40 | -0.030 |
| NGR32 | NGR40 | 0.069 |
| NGR33 | NGR40 | -0.029 |
| NGR34 | NGR40 | -0.020 |
| NGR35 | NGR40 | -0.005 |
| NGR36 | NGR40 | 0.006 |
| NGR37 | NGR40 | 0.062 |
| NGR38 | NGR40 | -0.018 |
| NGR39 | NGR40 | 0.015 |
| NGR1 | NGR41 | 0.016 |
| NGR2 | NGR41 | -0.031 |
| NGR3 | NGR41 | -0.046 |
| NGR4 | NGR41 | -0.038 |
| NGR5 | NGR41 | -0.054 |
| NGR6 | NGR41 | -0.084 |
| NGR7 | NGR41 | -0.049 |
| NGR8 | NGR41 | -0.146 |
| NGR9 | NGR41 | 0.040 |
| NGR10 | NGR41 | -0.038 |
| NGR11 | NGR41 | -0.059 |
| NGR12 | NGR41 | 0.088 |
| NGR13 | NGR41 | -0.075 |
| NGR14 | NGR41 | 0.054 |
| NGR15 | NGR41 | -0.059 |
| NGR16 | NGR41 | -0.073 |
| NGR17 | NGR41 | -0.004 |
| NGR18 | NGR41 | 0.082 |
| NGR19 | NGR41 | 0.011 |
| NGR20 | NGR41 | -0.023 |
| NGR21 | NGR41 | -0.068 |
| NGR22 | NGR41 | -0.024 |
| NGR23 | NGR41 | 0.060 |
| NGR24 | NGR41 | -0.060 |
| NGR25 | NGR41 | 0.016 |
| NGR26 | NGR41 | -0.086 |
| NGR27 | NGR41 | -0.049 |
| NGR28 | NGR41 | 0.019 |
| NGR29 | NGR41 | 0.049 |
| NGR30 | NGR41 | -0.033 |
| NGR31 | NGR41 | -0.024 |
| NGR32 | NGR41 | 0.128 |
| NGR33 | NGR41 | 0.001 |
| NGR34 | NGR41 | -0.075 |
| NGR35 | NGR41 | 0.160 |
| NGR36 | NGR41 | -0.049 |
| NGR37 | NGR41 | 0.030 |
| NGR38 | NGR41 | -0.049 |
| NGR39 | NGR41 | -0.040 |
| NGR40 | NGR41 | 0.080 |
| NGR1 | NGR42 | -0.025 |
| NGR2 | NGR42 | 0.067 |
| NGR3 | NGR42 | 0.199 |
| NGR4 | NGR42 | -0.035 |
| NGR5 | NGR42 | -0.054 |
| NGR6 | NGR42 | 0.289 |
| NGR7 | NGR42 | -0.038 |
| NGR8 | NGR42 | 0.218 |
| NGR9 | NGR42 | -0.060 |
| NGR10 | NGR42 | -0.027 |
| NGR11 | NGR42 | -0.056 |
| NGR12 | NGR42 | -0.017 |
| NGR13 | NGR42 | -0.016 |
| NGR14 | NGR42 | -0.018 |
| NGR15 | NGR42 | -0.056 |
| NGR16 | NGR42 | 0.005 |
| NGR17 | NGR42 | -0.021 |
| NGR18 | NGR42 | -0.028 |
| NGR19 | NGR42 | -0.019 |
| NGR20 | NGR42 | 0.013 |
| NGR21 | NGR42 | 0.044 |
| NGR22 | NGR42 | -0.079 |
| NGR23 | NGR42 | -0.050 |
| NGR24 | NGR42 | -0.007 |
| NGR25 | NGR42 | -0.077 |
| NGR26 | NGR42 | -0.083 |
| NGR27 | NGR42 | -0.038 |
| NGR28 | NGR42 | -0.075 |
| NGR29 | NGR42 | -0.020 |
| NGR30 | NGR42 | -0.042 |
| NGR31 | NGR42 | -0.060 |
| NGR32 | NGR42 | 0.071 |
| NGR33 | NGR42 | -0.109 |
| NGR34 | NGR42 | -0.016 |
| NGR35 | NGR42 | 0.010 |
| NGR36 | NGR42 | -0.038 |
| NGR37 | NGR42 | 0.009 |
| NGR38 | NGR42 | -0.049 |
| NGR39 | NGR42 | -0.038 |
| NGR40 | NGR42 | 0.011 |
| NGR41 | NGR42 | 0.036 |
| NGR1 | NGR43 | -0.014 |
| NGR2 | NGR43 | 0.015 |
| NGR3 | NGR43 | -0.121 |
| NGR4 | NGR43 | -0.008 |
| NGR5 | NGR43 | -0.032 |
| NGR6 | NGR43 | -0.084 |
| NGR7 | NGR43 | 0.041 |
| NGR8 | NGR43 | -0.146 |
| NGR9 | NGR43 | -0.083 |
| NGR10 | NGR43 | 0.008 |
| NGR11 | NGR43 | -0.026 |
| NGR12 | NGR43 | 0.004 |
| NGR13 | NGR43 | -0.011 |
| NGR14 | NGR43 | -0.006 |
| NGR15 | NGR43 | 0.023 |
| NGR16 | NGR43 | 0.018 |
| NGR17 | NGR43 | 0.029 |
| NGR18 | NGR43 | -0.007 |
| NGR19 | NGR43 | 0.013 |
| NGR20 | NGR43 | 0.011 |
| NGR21 | NGR43 | -0.013 |
| NGR22 | NGR43 | -0.025 |
| NGR23 | NGR43 | -0.042 |
| NGR24 | NGR43 | -0.055 |
| NGR25 | NGR43 | 0.017 |
| NGR26 | NGR43 | -0.005 |
| NGR27 | NGR43 | 0.041 |
| NGR28 | NGR43 | -0.074 |
| NGR29 | NGR43 | 0.045 |
| NGR30 | NGR43 | 0.058 |
| NGR31 | NGR43 | -0.039 |
| NGR32 | NGR43 | 0.001 |
| NGR33 | NGR43 | -0.053 |
| NGR34 | NGR43 | -0.011 |
| NGR35 | NGR43 | -0.005 |
| NGR36 | NGR43 | 0.041 |
| NGR37 | NGR43 | 0.053 |
| NGR38 | NGR43 | 0.017 |
| NGR39 | NGR43 | 0.006 |
| NGR40 | NGR43 | 0.067 |
| NGR41 | NGR43 | 0.012 |
| NGR42 | NGR43 | 0.002 |
| NGR1 | NGR44 | -0.042 |
| NGR2 | NGR44 | -0.010 |
| NGR3 | NGR44 | 0.033 |
| NGR4 | NGR44 | -0.105 |
| NGR5 | NGR44 | -0.105 |
| NGR6 | NGR44 | -0.095 |
| NGR7 | NGR44 | 0.077 |
| NGR8 | NGR44 | -0.164 |
| NGR9 | NGR44 | 0.126 |
| NGR10 | NGR44 | -0.020 |
| NGR11 | NGR44 | -0.062 |
| NGR12 | NGR44 | -0.111 |
| NGR13 | NGR44 | -0.065 |
| NGR14 | NGR44 | -0.062 |
| NGR15 | NGR44 | 0.059 |
| NGR16 | NGR44 | -0.012 |
| NGR17 | NGR44 | 0.066 |
| NGR18 | NGR44 | -0.062 |
| NGR19 | NGR44 | -0.007 |
| NGR20 | NGR44 | 0.002 |
| NGR21 | NGR44 | 0.063 |
| NGR22 | NGR44 | 0.665 |
| NGR23 | NGR44 | 0.240 |
| NGR24 | NGR44 | 0.175 |
| NGR25 | NGR44 | 0.187 |
| NGR26 | NGR44 | 0.153 |
| NGR27 | NGR44 | 0.032 |
| NGR28 | NGR44 | 0.274 |
| NGR29 | NGR44 | -0.062 |
| NGR30 | NGR44 | -0.039 |
| NGR31 | NGR44 | -0.048 |
| NGR32 | NGR44 | -0.072 |
| NGR33 | NGR44 | 0.096 |
| NGR34 | NGR44 | -0.065 |
| NGR35 | NGR44 | -0.111 |
| NGR36 | NGR44 | 0.077 |
| NGR37 | NGR44 | -0.063 |
| NGR38 | NGR44 | 0.054 |
| NGR39 | NGR44 | -0.012 |
| NGR40 | NGR44 | -0.049 |
| NGR41 | NGR44 | -0.095 |
| NGR42 | NGR44 | -0.092 |
| NGR43 | NGR44 | -0.013 |
| NGR1 | NGR45 | -0.025 |
| NGR2 | NGR45 | -0.007 |
| NGR3 | NGR45 | 0.035 |
| NGR4 | NGR45 | -0.008 |
| NGR5 | NGR45 | -0.054 |
| NGR6 | NGR45 | -0.006 |
| NGR7 | NGR45 | -0.007 |
| NGR8 | NGR45 | -0.068 |
| NGR9 | NGR45 | -0.083 |
| NGR10 | NGR45 | -0.015 |
| NGR11 | NGR45 | 0.519 |
| NGR12 | NGR45 | 0.004 |
| NGR13 | NGR45 | -0.033 |
| NGR14 | NGR45 | -0.006 |
| NGR15 | NGR45 | -0.026 |
| NGR16 | NGR45 | -0.030 |
| NGR17 | NGR45 | -0.019 |
| NGR18 | NGR45 | -0.007 |
| NGR19 | NGR45 | 0.002 |
| NGR20 | NGR45 | -0.038 |
| NGR21 | NGR45 | 0.043 |
| NGR22 | NGR45 | -0.073 |
| NGR23 | NGR45 | -0.090 |
| NGR24 | NGR45 | -0.078 |
| NGR25 | NGR45 | -0.031 |
| NGR26 | NGR45 | -0.053 |
| NGR27 | NGR45 | -0.007 |
| NGR28 | NGR45 | -0.074 |
| NGR29 | NGR45 | 0.022 |
| NGR30 | NGR45 | 0.009 |
| NGR31 | NGR45 | -0.039 |
| NGR32 | NGR45 | -0.022 |
| NGR33 | NGR45 | -0.090 |
| NGR34 | NGR45 | -0.033 |
| NGR35 | NGR45 | -0.005 |
| NGR36 | NGR45 | -0.007 |
| NGR37 | NGR45 | 0.030 |
| NGR38 | NGR45 | -0.031 |
| NGR39 | NGR45 | -0.017 |
| NGR40 | NGR45 | 0.045 |
| NGR41 | NGR45 | 0.012 |
| NGR42 | NGR45 | 0.002 |
| NGR43 | NGR45 | 0.045 |
| NGR44 | NGR45 | -0.062 |
| NGR1 | NGR46 | -0.060 |
| NGR2 | NGR46 | -0.042 |
| NGR3 | NGR46 | -0.121 |
| NGR4 | NGR46 | 0.009 |
| NGR5 | NGR46 | -0.077 |
| NGR6 | NGR46 | -0.102 |
| NGR7 | NGR46 | -0.024 |
| NGR8 | NGR46 | 0.036 |
| NGR9 | NGR46 | -0.097 |
| NGR10 | NGR46 | -0.050 |
| NGR11 | NGR46 | -0.065 |
| NGR12 | NGR46 | -0.041 |
| NGR13 | NGR46 | 0.000 |
| NGR14 | NGR46 | 0.127 |
| NGR15 | NGR46 | 0.194 |
| NGR16 | NGR46 | -0.047 |
| NGR17 | NGR46 | -0.007 |
| NGR18 | NGR46 | -0.031 |
| NGR19 | NGR46 | -0.010 |
| NGR20 | NGR46 | 0.002 |
| NGR21 | NGR46 | -0.061 |
| NGR22 | NGR46 | -0.060 |
| NGR23 | NGR46 | -0.013 |
| NGR24 | NGR46 | -0.066 |
| NGR25 | NGR46 | -0.006 |
| NGR26 | NGR46 | 0.053 |
| NGR27 | NGR46 | 0.003 |
| NGR28 | NGR46 | -0.023 |
| NGR29 | NGR46 | 0.006 |
| NGR30 | NGR46 | 0.020 |
| NGR31 | NGR46 | -0.044 |
| NGR32 | NGR46 | 0.010 |
| NGR33 | NGR46 | -0.070 |
| NGR34 | NGR46 | 0.005 |
| NGR35 | NGR46 | 0.029 |
| NGR36 | NGR46 | -0.024 |
| NGR37 | NGR46 | 0.007 |
| NGR38 | NGR46 | -0.036 |
| NGR39 | NGR46 | -0.024 |
| NGR40 | NGR46 | 0.019 |
| NGR41 | NGR46 | 0.021 |
| NGR42 | NGR46 | -0.027 |
| NGR43 | NGR46 | 0.019 |
| NGR44 | NGR46 | -0.070 |
| NGR45 | NGR46 | -0.004 |
| NGR1 | NGR47 | -0.055 |
| NGR2 | NGR47 | -0.062 |
| NGR3 | NGR47 | -0.073 |
| NGR4 | NGR47 | -0.041 |
| NGR5 | NGR47 | -0.005 |
| NGR6 | NGR47 | -0.119 |
| NGR7 | NGR47 | -0.044 |
| NGR8 | NGR47 | -0.146 |
| NGR9 | NGR47 | -0.136 |
| NGR10 | NGR47 | -0.044 |
| NGR11 | NGR47 | -0.085 |
| NGR12 | NGR47 | 0.373 |
| NGR13 | NGR47 | 0.058 |
| NGR14 | NGR47 | -0.084 |
| NGR15 | NGR47 | -0.053 |
| NGR16 | NGR47 | 0.027 |
| NGR17 | NGR47 | -0.044 |
| NGR18 | NGR47 | -0.075 |
| NGR19 | NGR47 | -0.055 |
| NGR20 | NGR47 | -0.008 |
| NGR21 | NGR47 | -0.033 |
| NGR22 | NGR47 | -0.014 |
| NGR23 | NGR47 | 0.045 |
| NGR24 | NGR47 | -0.044 |
| NGR25 | NGR47 | -0.022 |
| NGR26 | NGR47 | -0.035 |
| NGR27 | NGR47 | 0.011 |
| NGR28 | NGR47 | -0.066 |
| NGR29 | NGR47 | 0.028 |
| NGR30 | NGR47 | 0.087 |
| NGR31 | NGR47 | -0.085 |
| NGR32 | NGR47 | 0.054 |
| NGR33 | NGR47 | 0.398 |
| NGR34 | NGR47 | 0.167 |
| NGR35 | NGR47 | 0.010 |
| NGR36 | NGR47 | -0.044 |
| NGR37 | NGR47 | -0.022 |
| NGR38 | NGR47 | -0.044 |
| NGR39 | NGR47 | -0.053 |
| NGR40 | NGR47 | 0.028 |
| NGR41 | NGR47 | -0.004 |
| NGR42 | NGR47 | -0.050 |
| NGR43 | NGR47 | -0.022 |
| NGR44 | NGR47 | -0.098 |
| NGR45 | NGR47 | -0.045 |
| NGR46 | NGR47 | 0.014 |
| NGR1 | NGR48 | -0.002 |
| NGR2 | NGR48 | -0.026 |
| NGR3 | NGR48 | -0.037 |
| NGR4 | NGR48 | -0.008 |
| NGR5 | NGR48 | -0.073 |
| NGR6 | NGR48 | -0.093 |
| NGR7 | NGR48 | -0.007 |
| NGR8 | NGR48 | -0.146 |
| NGR9 | NGR48 | -0.101 |
| NGR10 | NGR48 | -0.033 |
| NGR11 | NGR48 | -0.026 |
| NGR12 | NGR48 | 0.070 |
| NGR13 | NGR48 | -0.033 |
| NGR14 | NGR48 | 0.059 |
| NGR15 | NGR48 | -0.026 |
| NGR16 | NGR48 | -0.030 |
| NGR17 | NGR48 | -0.019 |
| NGR18 | NGR48 | 0.067 |
| NGR19 | NGR48 | 0.035 |
| NGR20 | NGR48 | 0.017 |
| NGR21 | NGR48 | -0.044 |
| NGR22 | NGR48 | -0.073 |
| NGR23 | NGR48 | -0.036 |
| NGR24 | NGR48 | -0.033 |
| NGR25 | NGR48 | 0.024 |
| NGR26 | NGR48 | 0.001 |
| NGR27 | NGR48 | 0.047 |
| NGR28 | NGR48 | -0.019 |
| NGR29 | NGR48 | 0.013 |
| NGR30 | NGR48 | 0.064 |
| NGR31 | NGR48 | -0.048 |
| NGR32 | NGR48 | -0.040 |
| NGR33 | NGR48 | 0.006 |
| NGR34 | NGR48 | 0.076 |
| NGR35 | NGR48 | 0.070 |
| NGR36 | NGR48 | -0.007 |
| NGR37 | NGR48 | 0.012 |
| NGR38 | NGR48 | -0.031 |
| NGR39 | NGR48 | -0.026 |
| NGR40 | NGR48 | 0.026 |
| NGR41 | NGR48 | 0.077 |
| NGR42 | NGR48 | -0.016 |
| NGR43 | NGR48 | 0.035 |
| NGR44 | NGR48 | -0.062 |
| NGR45 | NGR48 | 0.035 |
| NGR46 | NGR48 | 0.051 |
| NGR47 | NGR48 | 0.064 |
| NGR1 | NGR49 | -0.014 |
| NGR2 | NGR49 | 0.015 |
| NGR3 | NGR49 | -0.121 |
| NGR4 | NGR49 | -0.026 |
| NGR5 | NGR49 | -0.032 |
| NGR6 | NGR49 | -0.075 |
| NGR7 | NGR49 | -0.003 |
| NGR8 | NGR49 | -0.146 |
| NGR9 | NGR49 | -0.083 |
| NGR10 | NGR49 | 0.008 |
| NGR11 | NGR49 | -0.044 |
| NGR12 | NGR49 | 0.004 |
| NGR13 | NGR49 | -0.029 |
| NGR14 | NGR49 | -0.006 |
| NGR15 | NGR49 | -0.003 |
| NGR16 | NGR49 | -0.026 |
| NGR17 | NGR49 | -0.015 |
| NGR18 | NGR49 | -0.017 |
| NGR19 | NGR49 | 0.004 |
| NGR20 | NGR49 | -0.034 |
| NGR21 | NGR49 | -0.022 |
| NGR22 | NGR49 | -0.060 |
| NGR23 | NGR49 | -0.086 |
| NGR24 | NGR49 | -0.065 |
| NGR25 | NGR49 | -0.027 |
| NGR26 | NGR49 | -0.049 |
| NGR27 | NGR49 | -0.003 |
| NGR28 | NGR49 | -0.092 |
| NGR29 | NGR49 | 0.045 |
| NGR30 | NGR49 | 0.013 |
| NGR31 | NGR49 | -0.030 |
| NGR32 | NGR49 | 0.010 |
| NGR33 | NGR49 | -0.088 |
| NGR34 | NGR49 | -0.029 |
| NGR35 | NGR49 | -0.014 |
| NGR36 | NGR49 | -0.003 |
| NGR37 | NGR49 | 0.053 |
| NGR38 | NGR49 | -0.027 |
| NGR39 | NGR49 | 0.015 |
| NGR40 | NGR49 | 0.076 |
| NGR41 | NGR49 | 0.021 |
| NGR42 | NGR49 | 0.002 |
| NGR43 | NGR49 | 0.058 |
| NGR44 | NGR49 | -0.058 |
| NGR45 | NGR49 | 0.035 |
| NGR46 | NGR49 | 0.019 |
| NGR47 | NGR49 | -0.032 |
| NGR48 | NGR49 | 0.017 |
| NGR1 | NGR50 | 0.000 |
| NGR2 | NGR50 | -0.006 |
| NGR3 | NGR50 | -0.121 |
| NGR4 | NGR50 | -0.075 |
| NGR5 | NGR50 | -0.050 |
| NGR6 | NGR50 | -0.075 |
| NGR7 | NGR50 | 0.082 |
| NGR8 | NGR50 | -0.146 |
| NGR9 | NGR50 | -0.036 |
| NGR10 | NGR50 | 0.011 |
| NGR11 | NGR50 | -0.075 |
| NGR12 | NGR50 | -0.057 |
| NGR13 | NGR50 | -0.034 |
| NGR14 | NGR50 | -0.074 |
| NGR15 | NGR50 | 0.064 |
| NGR16 | NGR50 | 0.018 |
| NGR17 | NGR50 | 0.128 |
| NGR18 | NGR50 | -0.066 |
| NGR19 | NGR50 | -0.002 |
| NGR20 | NGR50 | 0.064 |
| NGR21 | NGR50 | -0.072 |
| NGR22 | NGR50 | -0.027 |
| NGR23 | NGR50 | 0.028 |
| NGR24 | NGR50 | -0.044 |
| NGR25 | NGR50 | -0.063 |
| NGR26 | NGR50 | -0.009 |
| NGR27 | NGR50 | 0.037 |
| NGR28 | NGR50 | -0.021 |
| NGR29 | NGR50 | -0.044 |
| NGR30 | NGR50 | -0.009 |
| NGR31 | NGR50 | 0.070 |
| NGR32 | NGR50 | 0.047 |
| NGR33 | NGR50 | -0.021 |
| NGR34 | NGR50 | -0.034 |
| NGR35 | NGR50 | 0.034 |
| NGR36 | NGR50 | 0.082 |
| NGR37 | NGR50 | -0.034 |
| NGR38 | NGR50 | 0.082 |
| NGR39 | NGR50 | -0.007 |
| NGR40 | NGR50 | -0.044 |
| NGR41 | NGR50 | 0.015 |
| NGR42 | NGR50 | -0.030 |
| NGR43 | NGR50 | -0.009 |
| NGR44 | NGR50 | 0.028 |
| NGR45 | NGR50 | -0.057 |
| NGR46 | NGR50 | -0.016 |
| NGR47 | NGR50 | -0.044 |
| NGR48 | NGR50 | -0.057 |
| NGR49 | NGR50 | -0.053 |
| NGR1 | NGR51 | -0.015 |
| NGR2 | NGR51 | -0.052 |
| NGR3 | NGR51 | 0.100 |
| NGR4 | NGR51 | -0.039 |
| NGR5 | NGR51 | -0.073 |
| NGR6 | NGR51 | -0.050 |
| NGR7 | NGR51 | -0.052 |
| NGR8 | NGR51 | -0.068 |
| NGR9 | NGR51 | 0.027 |
| NGR10 | NGR51 | -0.033 |
| NGR11 | NGR51 | -0.052 |
| NGR12 | NGR51 | 0.070 |
| NGR13 | NGR51 | -0.052 |
| NGR14 | NGR51 | 0.033 |
| NGR15 | NGR51 | -0.070 |
| NGR16 | NGR51 | -0.049 |
| NGR17 | NGR51 | -0.064 |
| NGR18 | NGR51 | 0.109 |
| NGR19 | NGR51 | -0.001 |
| NGR20 | NGR51 | -0.082 |
| NGR21 | NGR51 | -0.002 |
| NGR22 | NGR51 | -0.099 |
| NGR23 | NGR51 | 0.046 |
| NGR24 | NGR51 | -0.032 |
| NGR25 | NGR51 | 0.079 |
| NGR26 | NGR51 | -0.079 |
| NGR27 | NGR51 | -0.052 |
| NGR28 | NGR51 | 0.037 |
| NGR29 | NGR51 | -0.009 |
| NGR30 | NGR51 | -0.009 |
| NGR31 | NGR51 | 0.116 |
| NGR32 | NGR51 | -0.066 |
| NGR33 | NGR51 | -0.061 |
| NGR34 | NGR51 | -0.052 |
| NGR35 | NGR51 | 0.060 |
| NGR36 | NGR51 | -0.052 |
| NGR37 | NGR51 | -0.014 |
| NGR38 | NGR51 | -0.075 |
| NGR39 | NGR51 | -0.061 |
| NGR40 | NGR51 | 0.000 |
| NGR41 | NGR51 | 0.128 |
| NGR42 | NGR51 | -0.042 |
| NGR43 | NGR51 | 0.000 |
| NGR44 | NGR51 | -0.088 |
| NGR45 | NGR51 | 0.078 |
| NGR46 | NGR51 | -0.048 |
| NGR47 | NGR51 | -0.063 |
| NGR48 | NGR51 | 0.075 |
| NGR49 | NGR51 | -0.009 |
| NGR50 | NGR51 | 0.215 |
| NGR1 | NGR52 | -0.005 |
| NGR2 | NGR52 | 0.024 |
| NGR3 | NGR52 | -0.121 |
| NGR4 | NGR52 | -0.060 |
| NGR5 | NGR52 | -0.023 |
| NGR6 | NGR52 | -0.075 |
| NGR7 | NGR52 | 0.006 |
| NGR8 | NGR52 | -0.146 |
| NGR9 | NGR52 | -0.074 |
| NGR10 | NGR52 | 0.017 |
| NGR11 | NGR52 | -0.035 |
| NGR12 | NGR52 | -0.029 |
| NGR13 | NGR52 | -0.020 |
| NGR14 | NGR52 | 0.548 |
| NGR15 | NGR52 | -0.003 |
| NGR16 | NGR52 | -0.038 |
| NGR17 | NGR52 | -0.006 |
| NGR18 | NGR52 | -0.007 |
| NGR19 | NGR52 | 0.013 |
| NGR20 | NGR52 | -0.024 |
| NGR21 | NGR52 | -0.013 |
| NGR22 | NGR52 | -0.022 |
| NGR23 | NGR52 | -0.098 |
| NGR24 | NGR52 | -0.055 |
| NGR25 | NGR52 | -0.060 |
| NGR26 | NGR52 | -0.040 |
| NGR27 | NGR52 | 0.006 |
| NGR28 | NGR52 | -0.083 |
| NGR29 | NGR52 | 0.061 |
| NGR30 | NGR52 | -0.020 |
| NGR31 | NGR52 | -0.030 |
| NGR32 | NGR52 | 0.069 |
| NGR33 | NGR52 | -0.029 |
| NGR34 | NGR52 | -0.020 |
| NGR35 | NGR52 | -0.048 |
| NGR36 | NGR52 | 0.006 |
| NGR37 | NGR52 | 0.019 |
| NGR38 | NGR52 | -0.018 |
| NGR39 | NGR52 | 0.015 |
| NGR40 | NGR52 | 0.093 |
| NGR41 | NGR52 | 0.037 |
| NGR42 | NGR52 | -0.010 |
| NGR43 | NGR52 | 0.024 |
| NGR44 | NGR52 | -0.049 |
| NGR45 | NGR52 | 0.002 |
| NGR46 | NGR52 | -0.024 |
| NGR47 | NGR52 | 0.006 |
| NGR48 | NGR52 | -0.017 |
| NGR49 | NGR52 | 0.034 |
| NGR50 | NGR52 | -0.044 |
| NGR51 | NGR52 | -0.042 |
| NGR1 | NGR53 | -0.009 |
| NGR2 | NGR53 | 0.008 |
| NGR3 | NGR53 | -0.121 |
| NGR4 | NGR53 | 0.002 |
| NGR5 | NGR53 | 0.041 |
| NGR6 | NGR53 | -0.100 |
| NGR7 | NGR53 | -0.011 |
| NGR8 | NGR53 | -0.146 |
| NGR9 | NGR53 | -0.090 |
| NGR10 | NGR53 | 0.026 |
| NGR11 | NGR53 | -0.052 |
| NGR12 | NGR53 | 0.077 |
| NGR13 | NGR53 | 0.044 |
| NGR14 | NGR53 | -0.014 |
| NGR15 | NGR53 | -0.029 |
| NGR16 | NGR53 | 0.047 |
| NGR17 | NGR53 | -0.023 |
| NGR18 | NGR53 | -0.024 |
| NGR19 | NGR53 | -0.004 |
| NGR20 | NGR53 | -0.041 |
| NGR21 | NGR53 | -0.029 |
| NGR22 | NGR53 | -0.077 |
| NGR23 | NGR53 | -0.013 |
| NGR24 | NGR53 | -0.059 |
| NGR25 | NGR53 | -0.034 |
| NGR26 | NGR53 | -0.057 |
| NGR27 | NGR53 | -0.011 |
| NGR28 | NGR53 | -0.100 |
| NGR29 | NGR53 | 0.041 |
| NGR30 | NGR53 | 0.087 |
| NGR31 | NGR53 | -0.042 |
| NGR32 | NGR53 | -0.007 |
| NGR33 | NGR53 | -0.069 |
| NGR34 | NGR53 | 0.044 |
| NGR35 | NGR53 | 0.059 |
| NGR36 | NGR53 | -0.011 |
| NGR37 | NGR53 | 0.045 |
| NGR38 | NGR53 | -0.034 |
| NGR39 | NGR53 | -0.011 |
| NGR40 | NGR53 | 0.060 |
| NGR41 | NGR53 | 0.004 |
| NGR42 | NGR53 | -0.005 |
| NGR43 | NGR53 | 0.050 |
| NGR44 | NGR53 | -0.065 |
| NGR45 | NGR53 | 0.028 |
| NGR46 | NGR53 | -0.007 |
| NGR47 | NGR53 | 0.032 |
| NGR48 | NGR53 | 0.009 |
| NGR49 | NGR53 | 0.050 |
| NGR50 | NGR53 | -0.034 |
| NGR51 | NGR53 | 0.009 |
| NGR52 | NGR53 | 0.017 |
| NGR1 | NGR54 | -0.002 |
| NGR2 | NGR54 | 0.095 |
| NGR3 | NGR54 | -0.063 |
| NGR4 | NGR54 | 0.239 |
| NGR5 | NGR54 | -0.059 |
| NGR6 | NGR54 | -0.068 |
| NGR7 | NGR54 | -0.060 |
| NGR8 | NGR54 | -0.117 |
| NGR9 | NGR54 | 0.044 |
| NGR10 | NGR54 | -0.054 |
| NGR11 | NGR54 | -0.078 |
| NGR12 | NGR54 | -0.001 |
| NGR13 | NGR54 | -0.001 |
| NGR14 | NGR54 | -0.059 |
| NGR15 | NGR54 | -0.069 |
| NGR16 | NGR54 | 0.039 |
| NGR17 | NGR54 | -0.066 |
| NGR18 | NGR54 | -0.035 |
| NGR19 | NGR54 | -0.026 |
| NGR20 | NGR54 | 0.002 |
| NGR21 | NGR54 | -0.001 |
| NGR22 | NGR54 | 0.002 |
| NGR23 | NGR54 | -0.095 |
| NGR24 | NGR54 | 0.065 |
| NGR25 | NGR54 | -0.049 |
| NGR26 | NGR54 | -0.106 |
| NGR27 | NGR54 | -0.060 |
| NGR28 | NGR54 | 0.072 |
| NGR29 | NGR54 | 0.036 |
| NGR30 | NGR54 | -0.007 |
| NGR31 | NGR54 | 0.117 |
| NGR32 | NGR54 | -0.003 |
| NGR33 | NGR54 | -0.043 |
| NGR34 | NGR54 | -0.050 |
| NGR35 | NGR54 | 0.015 |
| NGR36 | NGR54 | -0.060 |
| NGR37 | NGR54 | 0.047 |
| NGR38 | NGR54 | -0.014 |
| NGR39 | NGR54 | -0.060 |
| NGR40 | NGR54 | 0.051 |
| NGR41 | NGR54 | 0.030 |
| NGR42 | NGR54 | 0.042 |
| NGR43 | NGR54 | -0.008 |
| NGR44 | NGR54 | -0.114 |
| NGR45 | NGR54 | -0.008 |
| NGR46 | NGR54 | -0.016 |
| NGR47 | NGR54 | 0.009 |
| NGR48 | NGR54 | -0.017 |
| NGR49 | NGR54 | -0.008 |
| NGR50 | NGR54 | -0.085 |
| NGR51 | NGR54 | -0.039 |
| NGR52 | NGR54 | 0.009 |
| NGR53 | NGR54 | 0.011 |
| NGR1 | NGR55 | 0.100 |
| NGR2 | NGR55 | 0.146 |
| NGR3 | NGR55 | -0.006 |
| NGR4 | NGR55 | 0.038 |
| NGR5 | NGR55 | -0.032 |
| NGR6 | NGR55 | -0.017 |
| NGR7 | NGR55 | -0.027 |
| NGR8 | NGR55 | -0.088 |
| NGR9 | NGR55 | -0.083 |
| NGR10 | NGR55 | -0.016 |
| NGR11 | NGR55 | -0.068 |
| NGR12 | NGR55 | 0.004 |
| NGR13 | NGR55 | -0.053 |
| NGR14 | NGR55 | -0.030 |
| NGR15 | NGR55 | -0.045 |
| NGR16 | NGR55 | 0.037 |
| NGR17 | NGR55 | -0.027 |
| NGR18 | NGR55 | -0.040 |
| NGR19 | NGR55 | -0.020 |
| NGR20 | NGR55 | 0.041 |
| NGR21 | NGR55 | 0.041 |
| NGR22 | NGR55 | 0.045 |
| NGR23 | NGR55 | -0.086 |
| NGR24 | NGR55 | 0.135 |
| NGR25 | NGR55 | 0.019 |
| NGR26 | NGR55 | -0.073 |
| NGR27 | NGR55 | -0.027 |
| NGR28 | NGR55 | -0.104 |
| NGR29 | NGR55 | 0.012 |
| NGR30 | NGR55 | -0.010 |
| NGR31 | NGR55 | -0.072 |
| NGR32 | NGR55 | 0.001 |
| NGR33 | NGR55 | -0.098 |
| NGR34 | NGR55 | -0.053 |
| NGR35 | NGR55 | -0.014 |
| NGR36 | NGR55 | -0.027 |
| NGR37 | NGR55 | 0.167 |
| NGR38 | NGR55 | 0.088 |
| NGR39 | NGR55 | -0.027 |
| NGR40 | NGR55 | 0.043 |
| NGR41 | NGR55 | 0.012 |
| NGR42 | NGR55 | 0.077 |
| NGR43 | NGR55 | 0.034 |
| NGR44 | NGR55 | -0.082 |
| NGR45 | NGR55 | 0.012 |
| NGR46 | NGR55 | -0.011 |
| NGR47 | NGR55 | -0.041 |
| NGR48 | NGR55 | -0.007 |
| NGR49 | NGR55 | 0.034 |
| NGR50 | NGR55 | -0.053 |
| NGR51 | NGR55 | -0.033 |
| NGR52 | NGR55 | 0.001 |
| NGR53 | NGR55 | 0.027 |
| NGR54 | NGR55 | 0.183 |
| NGR1 | NGR56 | -0.039 |
| NGR2 | NGR56 | -0.016 |
| NGR3 | NGR56 | -0.121 |
| NGR4 | NGR56 | 0.107 |
| NGR5 | NGR56 | -0.028 |
| NGR6 | NGR56 | -0.106 |
| NGR7 | NGR56 | 0.002 |
| NGR8 | NGR56 | -0.146 |
| NGR9 | NGR56 | -0.114 |
| NGR10 | NGR56 | -0.011 |
| NGR11 | NGR56 | -0.039 |
| NGR12 | NGR56 | 0.008 |
| NGR13 | NGR56 | 0.111 |
| NGR14 | NGR56 | -0.038 |
| NGR15 | NGR56 | -0.016 |
| NGR16 | NGR56 | 0.015 |
| NGR17 | NGR56 | -0.010 |
| NGR18 | NGR56 | 0.020 |
| NGR19 | NGR56 | 0.040 |
| NGR20 | NGR56 | -0.028 |
| NGR21 | NGR56 | -0.035 |
| NGR22 | NGR56 | -0.064 |
| NGR23 | NGR56 | -0.045 |
| NGR24 | NGR56 | -0.071 |
| NGR25 | NGR56 | -0.021 |
| NGR26 | NGR56 | -0.044 |
| NGR27 | NGR56 | 0.002 |
| NGR28 | NGR56 | -0.037 |
| NGR29 | NGR56 | 0.029 |
| NGR30 | NGR56 | 0.055 |
| NGR31 | NGR56 | -0.005 |
| NGR32 | NGR56 | -0.031 |
| NGR33 | NGR56 | -0.063 |
| NGR34 | NGR56 | 0.012 |
| NGR35 | NGR56 | 0.058 |
| NGR36 | NGR56 | 0.002 |
| NGR37 | NGR56 | 0.021 |
| NGR38 | NGR56 | -0.021 |
| NGR39 | NGR56 | -0.016 |
| NGR40 | NGR56 | 0.036 |
| NGR41 | NGR56 | -0.020 |
| NGR42 | NGR56 | -0.029 |
| NGR43 | NGR56 | 0.045 |
| NGR44 | NGR56 | -0.052 |
| NGR45 | NGR56 | 0.022 |
| NGR46 | NGR56 | 0.055 |
| NGR47 | NGR56 | 0.000 |
| NGR48 | NGR56 | 0.022 |
| NGR49 | NGR56 | 0.026 |
| NGR50 | NGR56 | -0.034 |
| NGR51 | NGR56 | -0.009 |
| NGR52 | NGR56 | -0.007 |
| NGR53 | NGR56 | 0.055 |
| NGR54 | NGR56 | 0.049 |
| NGR55 | NGR56 | 0.003 |
| NGR1 | NGR57 | -0.014 |
| NGR2 | NGR57 | 0.015 |
| NGR3 | NGR57 | -0.026 |
| NGR4 | NGR57 | -0.001 |
| NGR5 | NGR57 | -0.032 |
| NGR6 | NGR57 | -0.084 |
| NGR7 | NGR57 | 0.015 |
| NGR8 | NGR57 | -0.146 |
| NGR9 | NGR57 | -0.083 |
| NGR10 | NGR57 | 0.008 |
| NGR11 | NGR57 | -0.026 |
| NGR12 | NGR57 | -0.038 |
| NGR13 | NGR57 | 0.133 |
| NGR14 | NGR57 | -0.006 |
| NGR15 | NGR57 | -0.003 |
| NGR16 | NGR57 | 0.018 |
| NGR17 | NGR57 | 0.003 |
| NGR18 | NGR57 | 0.017 |
| NGR19 | NGR57 | 0.038 |
| NGR20 | NGR57 | -0.015 |
| NGR21 | NGR57 | 0.082 |
| NGR22 | NGR57 | -0.025 |
| NGR23 | NGR57 | -0.042 |
| NGR24 | NGR57 | -0.055 |
| NGR25 | NGR57 | -0.051 |
| NGR26 | NGR57 | -0.031 |
| NGR27 | NGR57 | 0.015 |
| NGR28 | NGR57 | -0.049 |
| NGR29 | NGR57 | 0.002 |
| NGR30 | NGR57 | -0.011 |
| NGR31 | NGR57 | -0.014 |
| NGR32 | NGR57 | 0.095 |
| NGR33 | NGR57 | -0.079 |
| NGR34 | NGR57 | 0.084 |
| NGR35 | NGR57 | -0.023 |
| NGR36 | NGR57 | 0.015 |
| NGR37 | NGR57 | 0.010 |
| NGR38 | NGR57 | -0.009 |
| NGR39 | NGR57 | 0.006 |
| NGR40 | NGR57 | 0.024 |
| NGR41 | NGR57 | -0.031 |
| NGR42 | NGR57 | 0.028 |
| NGR43 | NGR57 | 0.024 |
| NGR44 | NGR57 | -0.039 |
| NGR45 | NGR57 | 0.002 |
| NGR46 | NGR57 | 0.001 |
| NGR47 | NGR57 | 0.004 |
| NGR48 | NGR57 | -0.007 |
| NGR49 | NGR57 | 0.015 |
| NGR50 | NGR57 | -0.034 |
| NGR51 | NGR57 | -0.042 |
| NGR52 | NGR57 | 0.024 |
| NGR53 | NGR57 | 0.008 |
| NGR54 | NGR57 | -0.026 |
| NGR55 | NGR57 | -0.009 |
| NGR56 | NGR57 | 0.052 |
| NGR1 | NGR58 | -0.027 |
| NGR2 | NGR58 | 0.144 |
| NGR3 | NGR58 | -0.026 |
| NGR4 | NGR58 | -0.041 |
| NGR5 | NGR58 | 0.023 |
| NGR6 | NGR58 | -0.100 |
| NGR7 | NGR58 | -0.029 |
| NGR8 | NGR58 | -0.146 |
| NGR9 | NGR58 | -0.109 |
| NGR10 | NGR58 | 0.008 |
| NGR11 | NGR58 | -0.070 |
| NGR12 | NGR58 | 0.016 |
| NGR13 | NGR58 | 0.120 |
| NGR14 | NGR58 | -0.032 |
| NGR15 | NGR58 | -0.048 |
| NGR16 | NGR58 | 0.141 |
| NGR17 | NGR58 | -0.041 |
| NGR18 | NGR58 | -0.042 |
| NGR19 | NGR58 | -0.022 |
| NGR20 | NGR58 | 0.027 |
| NGR21 | NGR58 | 0.133 |
| NGR22 | NGR58 | -0.069 |
| NGR23 | NGR58 | -0.005 |
| NGR24 | NGR58 | 0.077 |
| NGR25 | NGR58 | -0.096 |
| NGR26 | NGR58 | -0.075 |
| NGR27 | NGR58 | -0.029 |
| NGR28 | NGR58 | -0.118 |
| NGR29 | NGR58 | -0.020 |
| NGR30 | NGR58 | 0.025 |
| NGR31 | NGR58 | -0.061 |
| NGR32 | NGR58 | 0.070 |
| NGR33 | NGR58 | -0.088 |
| NGR34 | NGR58 | 0.120 |
| NGR35 | NGR58 | -0.002 |
| NGR36 | NGR58 | -0.029 |
| NGR37 | NGR58 | -0.016 |
| NGR38 | NGR58 | -0.053 |
| NGR39 | NGR58 | -0.029 |
| NGR40 | NGR58 | -0.002 |
| NGR41 | NGR58 | -0.057 |
| NGR42 | NGR58 | 0.089 |
| NGR43 | NGR58 | -0.011 |
| NGR44 | NGR58 | -0.084 |
| NGR45 | NGR58 | -0.033 |
| NGR46 | NGR58 | -0.068 |
| NGR47 | NGR58 | 0.040 |
| NGR48 | NGR58 | -0.052 |
| NGR49 | NGR58 | -0.011 |
| NGR50 | NGR58 | -0.053 |
| NGR51 | NGR58 | -0.052 |
| NGR52 | NGR58 | -0.002 |
| NGR53 | NGR58 | 0.062 |
| NGR54 | NGR58 | 0.105 |
| NGR55 | NGR58 | 0.120 |
| NGR56 | NGR58 | -0.007 |
| NGR57 | NGR58 | 0.084 |
| NGR1 | NGR59 | -0.051 |
| NGR2 | NGR59 | -0.022 |
| NGR3 | NGR59 | -0.026 |
| NGR4 | NGR59 | -0.020 |
| NGR5 | NGR59 | -0.069 |
| NGR6 | NGR59 | -0.093 |
| NGR7 | NGR59 | 0.023 |
| NGR8 | NGR59 | -0.146 |
| NGR9 | NGR59 | -0.120 |
| NGR10 | NGR59 | -0.029 |
| NGR11 | NGR59 | -0.044 |
| NGR12 | NGR59 | -0.075 |
| NGR13 | NGR59 | 0.115 |
| NGR14 | NGR59 | -0.043 |
| NGR15 | NGR59 | 0.458 |
| NGR16 | NGR59 | 0.026 |
| NGR17 | NGR59 | 0.011 |
| NGR18 | NGR59 | -0.010 |
| NGR19 | NGR59 | 0.010 |
| NGR20 | NGR59 | -0.008 |
| NGR21 | NGR59 | 0.054 |
| NGR22 | NGR59 | -0.008 |
| NGR23 | NGR59 | -0.034 |
| NGR24 | NGR59 | -0.083 |
| NGR25 | NGR59 | -0.044 |
| NGR26 | NGR59 | -0.023 |
| NGR27 | NGR59 | 0.023 |
| NGR28 | NGR59 | -0.067 |
| NGR29 | NGR59 | -0.016 |
| NGR30 | NGR59 | -0.003 |
| NGR31 | NGR59 | -0.023 |
| NGR32 | NGR59 | 0.068 |
| NGR33 | NGR59 | -0.062 |
| NGR34 | NGR59 | 0.065 |
| NGR35 | NGR59 | -0.051 |
| NGR36 | NGR59 | 0.023 |
| NGR37 | NGR59 | -0.027 |
| NGR38 | NGR59 | -0.001 |
| NGR39 | NGR59 | -0.003 |
| NGR40 | NGR59 | -0.003 |
| NGR41 | NGR59 | -0.059 |
| NGR42 | NGR59 | -0.009 |
| NGR43 | NGR59 | 0.023 |
| NGR44 | NGR59 | -0.032 |
| NGR45 | NGR59 | -0.026 |
| NGR46 | NGR59 | 0.219 |
| NGR47 | NGR59 | -0.006 |
| NGR48 | NGR59 | -0.026 |
| NGR49 | NGR59 | -0.003 |
| NGR50 | NGR59 | -0.027 |
| NGR51 | NGR59 | -0.070 |
| NGR52 | NGR59 | -0.003 |
| NGR53 | NGR59 | -0.029 |
| NGR54 | NGR59 | -0.044 |
| NGR55 | NGR59 | -0.045 |
| NGR56 | NGR59 | 0.033 |
| NGR57 | NGR59 | 0.116 |
| NGR58 | NGR59 | 0.047 |
| NGR1 | NGR60 | -0.036 |
| NGR2 | NGR60 | -0.020 |
| NGR3 | NGR60 | -0.121 |
| NGR4 | NGR60 | -0.030 |
| NGR5 | NGR60 | -0.041 |
| NGR6 | NGR60 | -0.110 |
| NGR7 | NGR60 | 0.025 |
| NGR8 | NGR60 | 0.399 |
| NGR9 | NGR60 | -0.118 |
| NGR10 | NGR60 | -0.002 |
| NGR11 | NGR60 | -0.061 |
| NGR12 | NGR60 | -0.005 |
| NGR13 | NGR60 | -0.020 |
| NGR14 | NGR60 | -0.041 |
| NGR15 | NGR60 | 0.016 |
| NGR16 | NGR60 | 0.028 |
| NGR17 | NGR60 | 0.014 |
| NGR18 | NGR60 | -0.042 |
| NGR19 | NGR60 | -0.022 |
| NGR20 | NGR60 | -0.050 |
| NGR21 | NGR60 | -0.048 |
| NGR22 | NGR60 | 0.018 |
| NGR23 | NGR60 | -0.077 |
| NGR24 | NGR60 | -0.078 |
| NGR25 | NGR60 | -0.044 |
| NGR26 | NGR60 | -0.066 |
| NGR27 | NGR60 | -0.020 |
| NGR28 | NGR60 | -0.109 |
| NGR29 | NGR60 | 0.082 |
| NGR30 | NGR60 | 0.023 |
| NGR31 | NGR60 | -0.052 |
| NGR32 | NGR60 | 0.025 |
| NGR33 | NGR60 | -0.042 |
| NGR34 | NGR60 | -0.020 |
| NGR35 | NGR60 | -0.014 |
| NGR36 | NGR60 | 0.025 |
| NGR37 | NGR60 | 0.017 |
| NGR38 | NGR60 | 0.002 |
| NGR39 | NGR60 | -0.020 |
| NGR40 | NGR60 | 0.091 |
| NGR41 | NGR60 | 0.036 |
| NGR42 | NGR60 | -0.033 |
| NGR43 | NGR60 | 0.032 |
| NGR44 | NGR60 | -0.029 |
| NGR45 | NGR60 | 0.009 |
| NGR46 | NGR60 | -0.007 |
| NGR47 | NGR60 | 0.028 |
| NGR48 | NGR60 | 0.000 |
| NGR49 | NGR60 | 0.032 |
| NGR50 | NGR60 | 0.002 |
| NGR51 | NGR60 | -0.009 |
| NGR52 | NGR60 | 0.048 |
| NGR53 | NGR60 | 0.041 |
| NGR54 | NGR60 | 0.029 |
| NGR55 | NGR60 | -0.001 |
| NGR56 | NGR60 | 0.023 |
| NGR57 | NGR60 | -0.011 |
| NGR58 | NGR60 | -0.020 |
| NGR59 | NGR60 | -0.029 |
| NGR1 | NGR61 | 0.018 |
| NGR2 | NGR61 | 0.013 |
| NGR3 | NGR61 | -0.121 |
| NGR4 | NGR61 | -0.003 |
| NGR5 | NGR61 | -0.009 |
| NGR6 | NGR61 | -0.066 |
| NGR7 | NGR61 | 0.011 |
| NGR8 | NGR61 | -0.146 |
| NGR9 | NGR61 | -0.018 |
| NGR10 | NGR61 | 0.029 |
| NGR11 | NGR61 | -0.075 |
| NGR12 | NGR61 | -0.016 |
| NGR13 | NGR61 | 0.038 |
| NGR14 | NGR61 | -0.056 |
| NGR15 | NGR61 | -0.007 |
| NGR16 | NGR61 | -0.030 |
| NGR17 | NGR61 | 0.056 |
| NGR18 | NGR61 | -0.032 |
| NGR19 | NGR61 | 0.032 |
| NGR20 | NGR61 | 0.038 |
| NGR21 | NGR61 | -0.062 |
| NGR22 | NGR61 | -0.098 |
| NGR23 | NGR61 | 0.025 |
| NGR24 | NGR61 | -0.035 |
| NGR25 | NGR61 | -0.089 |
| NGR26 | NGR61 | -0.035 |
| NGR27 | NGR61 | 0.011 |
| NGR28 | NGR61 | 0.004 |
| NGR29 | NGR61 | -0.034 |
| NGR30 | NGR61 | -0.012 |
| NGR31 | NGR61 | 0.104 |
| NGR32 | NGR61 | 0.066 |
| NGR33 | NGR61 | -0.024 |
| NGR34 | NGR61 | -0.012 |
| NGR35 | NGR61 | 0.091 |
| NGR36 | NGR61 | 0.011 |
| NGR37 | NGR61 | -0.016 |
| NGR38 | NGR61 | 0.011 |
| NGR39 | NGR61 | 0.002 |
| NGR40 | NGR61 | -0.025 |
| NGR41 | NGR61 | 0.034 |
| NGR42 | NGR61 | -0.012 |
| NGR43 | NGR61 | -0.025 |
| NGR44 | NGR61 | -0.044 |
| NGR45 | NGR61 | -0.048 |
| NGR46 | NGR61 | 0.009 |
| NGR47 | NGR61 | -0.021 |
| NGR48 | NGR61 | -0.057 |
| NGR49 | NGR61 | -0.034 |
| NGR50 | NGR61 | 0.211 |
| NGR51 | NGR61 | 0.043 |
| NGR52 | NGR61 | -0.025 |
| NGR53 | NGR61 | 0.007 |
| NGR54 | NGR61 | -0.028 |
| NGR55 | NGR61 | -0.034 |
| NGR56 | NGR61 | 0.038 |
| NGR57 | NGR61 | 0.000 |
| NGR58 | NGR61 | -0.012 |
| NGR59 | NGR61 | -0.028 |
| NGR60 | NGR61 | -0.034 |
| NGR1 | NGR62 | -0.033 |
| NGR2 | NGR62 | -0.003 |
| NGR3 | NGR62 | -0.026 |
| NGR4 | NGR62 | 0.048 |
| NGR5 | NGR62 | -0.050 |
| NGR6 | NGR62 | -0.093 |
| NGR7 | NGR62 | 0.015 |
| NGR8 | NGR62 | -0.146 |
| NGR9 | NGR62 | -0.101 |
| NGR10 | NGR62 | -0.011 |
| NGR11 | NGR62 | -0.026 |
| NGR12 | NGR62 | -0.057 |
| NGR13 | NGR62 | 0.183 |
| NGR14 | NGR62 | -0.025 |
| NGR15 | NGR62 | -0.003 |
| NGR16 | NGR62 | 0.018 |
| NGR17 | NGR62 | 0.003 |
| NGR18 | NGR62 | 0.033 |
| NGR19 | NGR62 | 0.053 |
| NGR20 | NGR62 | -0.015 |
| NGR21 | NGR62 | 0.073 |
| NGR22 | NGR62 | -0.025 |
| NGR23 | NGR62 | -0.042 |
| NGR24 | NGR62 | -0.065 |
| NGR25 | NGR62 | -0.051 |
| NGR26 | NGR62 | -0.031 |
| NGR27 | NGR62 | 0.015 |
| NGR28 | NGR62 | -0.024 |
| NGR29 | NGR62 | -0.007 |
| NGR30 | NGR62 | -0.011 |
| NGR31 | NGR62 | 0.002 |
| NGR32 | NGR62 | 0.077 |
| NGR33 | NGR62 | -0.079 |
| NGR34 | NGR62 | 0.084 |
| NGR35 | NGR62 | -0.007 |
| NGR36 | NGR62 | 0.015 |
| NGR37 | NGR62 | -0.009 |
| NGR38 | NGR62 | -0.009 |
| NGR39 | NGR62 | -0.003 |
| NGR40 | NGR62 | 0.006 |
| NGR41 | NGR62 | -0.049 |
| NGR42 | NGR62 | 0.010 |
| NGR43 | NGR62 | 0.015 |
| NGR44 | NGR62 | -0.039 |
| NGR45 | NGR62 | -0.007 |
| NGR46 | NGR62 | 0.026 |
| NGR47 | NGR62 | 0.004 |
| NGR48 | NGR62 | -0.007 |
| NGR49 | NGR62 | -0.003 |
| NGR50 | NGR62 | -0.034 |
| NGR51 | NGR62 | -0.052 |
| NGR52 | NGR62 | 0.006 |
| NGR53 | NGR62 | -0.011 |
| NGR54 | NGR62 | -0.010 |
| NGR55 | NGR62 | -0.027 |
| NGR56 | NGR62 | 0.101 |
| NGR57 | NGR62 | 0.159 |
| NGR58 | NGR62 | 0.065 |
| NGR59 | NGR62 | 0.141 |
| NGR60 | NGR62 | -0.020 |
| NGR61 | NGR62 | 0.015 |
| NGR1 | NGR63 | -0.046 |
| NGR2 | NGR63 | -0.029 |
| NGR3 | NGR63 | -0.121 |
| NGR4 | NGR63 | -0.064 |
| NGR5 | NGR63 | -0.018 |
| NGR6 | NGR63 | -0.119 |
| NGR7 | NGR63 | 0.015 |
| NGR8 | NGR63 | -0.146 |
| NGR9 | NGR63 | -0.127 |
| NGR10 | NGR63 | -0.011 |
| NGR11 | NGR63 | -0.052 |
| NGR12 | NGR63 | -0.025 |
| NGR13 | NGR63 | 0.021 |
| NGR14 | NGR63 | -0.051 |
| NGR15 | NGR63 | -0.003 |
| NGR16 | NGR63 | 0.029 |
| NGR17 | NGR63 | 0.003 |
| NGR18 | NGR63 | 0.321 |
| NGR19 | NGR63 | -0.022 |
| NGR20 | NGR63 | 0.012 |
| NGR21 | NGR63 | -0.048 |
| NGR22 | NGR63 | -0.072 |
| NGR23 | NGR63 | -0.004 |
| NGR24 | NGR63 | -0.050 |
| NGR25 | NGR63 | -0.024 |
| NGR26 | NGR63 | -0.003 |
| NGR27 | NGR63 | 0.042 |
| NGR28 | NGR63 | -0.072 |
| NGR29 | NGR63 | -0.020 |
| NGR30 | NGR63 | 0.074 |
| NGR31 | NGR63 | -0.061 |
| NGR32 | NGR63 | -0.044 |
| NGR33 | NGR63 | -0.039 |
| NGR34 | NGR63 | 0.076 |
| NGR35 | NGR63 | -0.025 |
| NGR36 | NGR63 | 0.015 |
| NGR37 | NGR63 | -0.034 |
| NGR38 | NGR63 | -0.009 |
| NGR39 | NGR63 | -0.029 |
| NGR40 | NGR63 | -0.020 |
| NGR41 | NGR63 | -0.075 |
| NGR42 | NGR63 | -0.064 |
| NGR43 | NGR63 | 0.015 |
| NGR44 | NGR63 | -0.039 |
| NGR45 | NGR63 | -0.033 |
| NGR46 | NGR63 | -0.023 |
| NGR47 | NGR63 | 0.043 |
| NGR48 | NGR63 | 0.021 |
| NGR49 | NGR63 | -0.029 |
| NGR50 | NGR63 | -0.009 |
| NGR51 | NGR63 | -0.052 |
| NGR52 | NGR63 | -0.020 |
| NGR53 | NGR63 | 0.021 |
| NGR54 | NGR63 | -0.073 |
| NGR55 | NGR63 | -0.053 |
| NGR56 | NGR63 | -0.011 |
| NGR57 | NGR63 | -0.011 |
| NGR58 | NGR63 | 0.003 |
| NGR59 | NGR63 | -0.003 |
| NGR60 | NGR63 | -0.020 |
| NGR61 | NGR63 | -0.034 |
| NGR62 | NGR63 | -0.011 |
| NGR1 | NGR64 | -0.015 |
| NGR2 | NGR64 | -0.075 |
| NGR3 | NGR64 | -0.037 |
| NGR4 | NGR64 | -0.003 |
| NGR5 | NGR64 | -0.018 |
| NGR6 | NGR64 | -0.119 |
| NGR7 | NGR64 | -0.057 |
| NGR8 | NGR64 | -0.146 |
| NGR9 | NGR64 | -0.082 |
| NGR10 | NGR64 | -0.057 |
| NGR11 | NGR64 | -0.075 |
| NGR12 | NGR64 | 0.082 |
| NGR13 | NGR64 | 0.047 |
| NGR14 | NGR64 | 0.009 |
| NGR15 | NGR64 | -0.075 |
| NGR16 | NGR64 | -0.021 |
| NGR17 | NGR64 | -0.012 |
| NGR18 | NGR64 | 0.042 |
| NGR19 | NGR64 | 0.010 |
| NGR20 | NGR64 | -0.003 |
| NGR21 | NGR64 | -0.094 |
| NGR22 | NGR64 | -0.121 |
| NGR23 | NGR64 | 0.061 |
| NGR24 | NGR64 | -0.085 |
| NGR25 | NGR64 | -0.084 |
| NGR26 | NGR64 | -0.075 |
| NGR27 | NGR64 | -0.030 |
| NGR28 | NGR64 | -0.014 |
| NGR29 | NGR64 | -0.066 |
| NGR30 | NGR64 | 0.025 |
| NGR31 | NGR64 | -0.060 |
| NGR32 | NGR64 | 0.025 |
| NGR33 | NGR64 | -0.011 |
| NGR34 | NGR64 | 0.052 |
| NGR35 | NGR64 | 0.197 |
| NGR36 | NGR64 | -0.057 |
| NGR37 | NGR64 | -0.057 |
| NGR38 | NGR64 | -0.057 |
| NGR39 | NGR64 | -0.075 |
| NGR40 | NGR64 | -0.066 |
| NGR41 | NGR64 | 0.099 |
| NGR42 | NGR64 | -0.030 |
| NGR43 | NGR64 | -0.057 |
| NGR44 | NGR64 | -0.111 |
| NGR45 | NGR64 | -0.057 |
| NGR46 | NGR64 | 0.013 |
| NGR47 | NGR64 | 0.043 |
| NGR48 | NGR64 | 0.081 |
| NGR49 | NGR64 | -0.075 |
| NGR50 | NGR64 | 0.034 |
| NGR51 | NGR64 | 0.008 |
| NGR52 | NGR64 | -0.066 |
| NGR53 | NGR64 | -0.002 |
| NGR54 | NGR64 | -0.037 |
| NGR55 | NGR64 | -0.075 |
| NGR56 | NGR64 | 0.015 |
| NGR57 | NGR64 | -0.032 |
| NGR58 | NGR64 | -0.021 |
| NGR59 | NGR64 | -0.051 |
| NGR60 | NGR64 | -0.066 |
| NGR61 | NGR64 | 0.081 |
| NGR62 | NGR64 | -0.007 |
| NGR63 | NGR64 | 0.002 |
| NGR1 | NGR65 | -0.033 |
| NGR2 | NGR65 | -0.022 |
| NGR3 | NGR65 | -0.121 |
| NGR4 | NGR65 | 0.135 |
| NGR5 | NGR65 | 0.012 |
| NGR6 | NGR65 | -0.110 |
| NGR7 | NGR65 | -0.022 |
| NGR8 | NGR65 | -0.146 |
| NGR9 | NGR65 | -0.025 |
| NGR10 | NGR65 | -0.004 |
| NGR11 | NGR65 | -0.052 |
| NGR12 | NGR65 | 0.059 |
| NGR13 | NGR65 | 0.082 |
| NGR14 | NGR65 | -0.032 |
| NGR15 | NGR65 | -0.040 |
| NGR16 | NGR65 | 0.036 |
| NGR17 | NGR65 | -0.034 |
| NGR18 | NGR65 | -0.008 |
| NGR19 | NGR65 | 0.006 |
| NGR20 | NGR65 | -0.052 |
| NGR21 | NGR65 | -0.050 |
| NGR22 | NGR65 | -0.088 |
| NGR23 | NGR65 | -0.024 |
| NGR24 | NGR65 | -0.080 |
| NGR25 | NGR65 | -0.046 |
| NGR26 | NGR65 | -0.068 |
| NGR27 | NGR65 | -0.022 |
| NGR28 | NGR65 | 0.009 |
| NGR29 | NGR65 | 0.021 |
| NGR30 | NGR65 | 0.075 |
| NGR31 | NGR65 | 0.057 |
| NGR32 | NGR65 | -0.036 |
| NGR33 | NGR65 | -0.075 |
| NGR34 | NGR65 | 0.033 |
| NGR35 | NGR65 | 0.075 |
| NGR36 | NGR65 | -0.022 |
| NGR37 | NGR65 | 0.015 |
| NGR38 | NGR65 | -0.046 |
| NGR39 | NGR65 | -0.031 |
| NGR40 | NGR65 | 0.030 |
| NGR41 | NGR65 | -0.014 |
| NGR42 | NGR65 | -0.024 |
| NGR43 | NGR65 | 0.030 |
| NGR44 | NGR65 | -0.077 |
| NGR45 | NGR65 | 0.019 |
| NGR46 | NGR65 | 0.006 |
| NGR47 | NGR65 | 0.021 |
| NGR48 | NGR65 | 0.009 |
| NGR49 | NGR65 | 0.021 |
| NGR50 | NGR65 | -0.046 |
| NGR51 | NGR65 | 0.000 |
| NGR52 | NGR65 | -0.013 |
| NGR53 | NGR65 | 0.094 |
| NGR54 | NGR65 | 0.110 |
| NGR55 | NGR65 | -0.003 |
| NGR56 | NGR65 | 0.093 |
| NGR57 | NGR65 | 0.012 |
| NGR58 | NGR65 | 0.033 |
| NGR59 | NGR65 | -0.016 |
| NGR60 | NGR65 | 0.021 |
| NGR61 | NGR65 | 0.011 |
| NGR62 | NGR65 | 0.028 |
| NGR63 | NGR65 | 0.010 |
| NGR64 | NGR65 | 0.023 |
| NGR1 | NGR66 | 0.007 |
| NGR2 | NGR66 | 0.014 |
| NGR3 | NGR66 | -0.121 |
| NGR4 | NGR66 | 0.176 |
| NGR5 | NGR66 | -0.032 |
| NGR6 | NGR66 | -0.066 |
| NGR7 | NGR66 | 0.012 |
| NGR8 | NGR66 | -0.146 |
| NGR9 | NGR66 | 0.104 |
| NGR10 | NGR66 | 0.031 |
| NGR11 | NGR66 | -0.052 |
| NGR12 | NGR66 | -0.016 |
| NGR13 | NGR66 | 0.039 |
| NGR14 | NGR66 | -0.032 |
| NGR15 | NGR66 | -0.006 |
| NGR16 | NGR66 | -0.029 |
| NGR17 | NGR66 | 0.000 |
| NGR18 | NGR66 | -0.008 |
| NGR19 | NGR66 | 0.044 |
| NGR20 | NGR66 | -0.018 |
| NGR21 | NGR66 | -0.061 |
| NGR22 | NGR66 | -0.121 |
| NGR23 | NGR66 | -0.089 |
| NGR24 | NGR66 | -0.045 |
| NGR25 | NGR66 | -0.100 |
| NGR26 | NGR66 | -0.034 |
| NGR27 | NGR66 | 0.012 |
| NGR28 | NGR66 | 0.138 |
| NGR29 | NGR66 | -0.033 |
| NGR30 | NGR66 | -0.011 |
| NGR31 | NGR66 | 0.186 |
| NGR32 | NGR66 | -0.048 |
| NGR33 | NGR66 | -0.035 |
| NGR34 | NGR66 | -0.011 |
| NGR35 | NGR66 | 0.000 |
| NGR36 | NGR66 | 0.012 |
| NGR37 | NGR66 | -0.038 |
| NGR38 | NGR66 | -0.011 |
| NGR39 | NGR66 | 0.003 |
| NGR40 | NGR66 | -0.024 |
| NGR41 | NGR66 | -0.057 |
| NGR42 | NGR66 | -0.045 |
| NGR43 | NGR66 | -0.024 |
| NGR44 | NGR66 | -0.042 |
| NGR45 | NGR66 | -0.024 |
| NGR46 | NGR66 | -0.048 |
| NGR47 | NGR66 | -0.043 |
| NGR48 | NGR66 | -0.033 |
| NGR49 | NGR66 | -0.033 |
| NGR50 | NGR66 | -0.011 |
| NGR51 | NGR66 | -0.042 |
| NGR52 | NGR66 | -0.024 |
| NGR53 | NGR66 | 0.008 |
| NGR54 | NGR66 | 0.152 |
| NGR55 | NGR66 | -0.057 |
| NGR56 | NGR66 | 0.039 |
| NGR57 | NGR66 | 0.001 |
| NGR58 | NGR66 | -0.011 |
| NGR59 | NGR66 | -0.027 |
| NGR60 | NGR66 | -0.033 |
| NGR61 | NGR66 | 0.045 |
| NGR62 | NGR66 | 0.016 |
| NGR63 | NGR66 | -0.033 |
| NGR64 | NGR66 | -0.009 |
| NGR65 | NGR66 | 0.107 |
| NGR1 | NGR67 | 0.027 |
| NGR2 | NGR67 | -0.052 |
| NGR3 | NGR67 | -0.049 |
| NGR4 | NGR67 | -0.055 |
| NGR5 | NGR67 | -0.007 |
| NGR6 | NGR67 | -0.100 |
| NGR7 | NGR67 | -0.071 |
| NGR8 | NGR67 | -0.136 |
| NGR9 | NGR67 | 0.046 |
| NGR10 | NGR67 | -0.034 |
| NGR11 | NGR67 | -0.082 |
| NGR12 | NGR67 | 0.040 |
| NGR13 | NGR67 | -0.016 |
| NGR14 | NGR67 | -0.021 |
| NGR15 | NGR67 | -0.089 |
| NGR16 | NGR67 | -0.035 |
| NGR17 | NGR67 | -0.077 |
| NGR18 | NGR67 | 0.409 |
| NGR19 | NGR67 | -0.037 |
| NGR20 | NGR67 | -0.095 |
| NGR21 | NGR67 | -0.071 |
| NGR22 | NGR67 | -0.052 |
| NGR23 | NGR67 | 0.091 |
| NGR24 | NGR67 | 0.011 |
| NGR25 | NGR67 | 0.071 |
| NGR26 | NGR67 | -0.080 |
| NGR27 | NGR67 | -0.071 |
| NGR28 | NGR67 | 0.031 |
| NGR29 | NGR67 | -0.062 |
| NGR30 | NGR67 | -0.016 |
| NGR31 | NGR67 | -0.014 |
| NGR32 | NGR67 | -0.055 |
| NGR33 | NGR67 | -0.054 |
| NGR34 | NGR67 | -0.016 |
| NGR35 | NGR67 | 0.021 |
| NGR36 | NGR67 | -0.071 |
| NGR37 | NGR67 | 0.011 |
| NGR38 | NGR67 | -0.025 |
| NGR39 | NGR67 | -0.071 |
| NGR40 | NGR67 | -0.043 |
| NGR41 | NGR67 | 0.044 |
| NGR42 | NGR67 | -0.070 |
| NGR43 | NGR67 | -0.052 |
| NGR44 | NGR67 | -0.088 |
| NGR45 | NGR67 | -0.064 |
| NGR46 | NGR67 | -0.104 |
| NGR47 | NGR67 | -0.037 |
| NGR48 | NGR67 | -0.040 |
| NGR49 | NGR67 | -0.052 |
| NGR50 | NGR67 | -0.083 |
| NGR51 | NGR67 | 0.114 |
| NGR52 | NGR67 | -0.043 |
| NGR53 | NGR67 | 0.021 |
| NGR54 | NGR67 | -0.046 |
| NGR55 | NGR67 | -0.007 |
| NGR56 | NGR67 | -0.048 |
| NGR57 | NGR67 | -0.052 |
| NGR58 | NGR67 | 0.002 |
| NGR59 | NGR67 | -0.089 |
| NGR60 | NGR67 | -0.062 |
| NGR61 | NGR67 | -0.042 |
| NGR62 | NGR67 | -0.071 |
| NGR63 | NGR67 | 0.324 |
| NGR64 | NGR67 | 0.003 |
| NGR65 | NGR67 | -0.003 |
| NGR66 | NGR67 | -0.041 |
| NGR1 | NGR68 | -0.010 |
| NGR2 | NGR68 | 0.009 |
| NGR3 | NGR68 | -0.125 |
| NGR4 | NGR68 | -0.050 |
| NGR5 | NGR68 | -0.005 |
| NGR6 | NGR68 | -0.080 |
| NGR7 | NGR68 | 0.051 |
| NGR8 | NGR68 | -0.155 |
| NGR9 | NGR68 | -0.082 |
| NGR10 | NGR68 | 0.025 |
| NGR11 | NGR68 | -0.061 |
| NGR12 | NGR68 | -0.011 |
| NGR13 | NGR68 | 0.035 |
| NGR14 | NGR68 | -0.060 |
| NGR15 | NGR68 | 0.033 |
| NGR16 | NGR68 | 0.042 |
| NGR17 | NGR68 | 0.039 |
| NGR18 | NGR68 | -0.052 |
| NGR19 | NGR68 | 0.012 |
| NGR20 | NGR68 | 0.048 |
| NGR21 | NGR68 | -0.048 |
| NGR22 | NGR68 | -0.072 |
| NGR23 | NGR68 | 0.019 |
| NGR24 | NGR68 | -0.005 |
| NGR25 | NGR68 | -0.024 |
| NGR26 | NGR68 | 0.042 |
| NGR27 | NGR68 | 0.079 |
| NGR28 | NGR68 | -0.027 |
| NGR29 | NGR68 | -0.029 |
| NGR30 | NGR68 | 0.088 |
| NGR31 | NGR68 | -0.025 |
| NGR32 | NGR68 | -0.053 |
| NGR33 | NGR68 | 0.029 |
| NGR34 | NGR68 | 0.089 |
| NGR35 | NGR68 | -0.011 |
| NGR36 | NGR68 | 0.051 |
| NGR37 | NGR68 | -0.044 |
| NGR38 | NGR68 | 0.028 |
| NGR39 | NGR68 | 0.007 |
| NGR40 | NGR68 | -0.029 |
| NGR41 | NGR68 | -0.085 |
| NGR42 | NGR68 | -0.073 |
| NGR43 | NGR68 | 0.006 |
| NGR44 | NGR68 | 0.006 |
| NGR45 | NGR68 | -0.043 |
| NGR46 | NGR68 | -0.032 |
| NGR47 | NGR68 | 0.056 |
| NGR48 | NGR68 | 0.012 |
| NGR49 | NGR68 | -0.039 |
| NGR50 | NGR68 | 0.028 |
| NGR51 | NGR68 | -0.061 |
| NGR52 | NGR68 | -0.029 |
| NGR53 | NGR68 | 0.035 |
| NGR54 | NGR68 | -0.059 |
| NGR55 | NGR68 | -0.062 |
| NGR56 | NGR68 | 0.003 |
| NGR57 | NGR68 | -0.020 |
| NGR58 | NGR68 | 0.016 |
| NGR59 | NGR68 | -0.013 |
| NGR60 | NGR68 | -0.029 |
| NGR61 | NGR68 | 0.024 |
| NGR62 | NGR68 | -0.020 |
| NGR63 | NGR68 | 0.065 |
| NGR64 | NGR68 | 0.016 |
| NGR65 | NGR68 | 0.023 |
| NGR66 | NGR68 | 0.026 |
| NGR67 | NGR68 | 0.202 |
| NGR1 | NGR69 | 0.003 |
| NGR2 | NGR69 | -0.033 |
| NGR3 | NGR69 | -0.046 |
| NGR4 | NGR69 | -0.059 |
| NGR5 | NGR69 | 0.000 |
| NGR6 | NGR69 | -0.110 |
| NGR7 | NGR69 | -0.052 |
| NGR8 | NGR69 | -0.164 |
| NGR9 | NGR69 | -0.090 |
| NGR10 | NGR69 | -0.015 |
| NGR11 | NGR69 | -0.070 |
| NGR12 | NGR69 | 0.100 |
| NGR13 | NGR69 | 0.003 |
| NGR14 | NGR69 | 0.051 |
| NGR15 | NGR69 | -0.070 |
| NGR16 | NGR69 | -0.016 |
| NGR17 | NGR69 | -0.064 |
| NGR18 | NGR69 | 0.041 |
| NGR19 | NGR69 | 0.009 |
| NGR20 | NGR69 | -0.082 |
| NGR21 | NGR69 | -0.052 |
| NGR22 | NGR69 | -0.121 |
| NGR23 | NGR69 | -0.057 |
| NGR24 | NGR69 | -0.082 |
| NGR25 | NGR69 | -0.100 |
| NGR26 | NGR69 | -0.079 |
| NGR27 | NGR69 | -0.052 |
| NGR28 | NGR69 | -0.100 |
| NGR29 | NGR69 | -0.043 |
| NGR30 | NGR69 | 0.003 |
| NGR31 | NGR69 | -0.061 |
| NGR32 | NGR69 | -0.048 |
| NGR33 | NGR69 | -0.039 |
| NGR34 | NGR69 | 0.003 |
| NGR35 | NGR69 | 0.081 |
| NGR36 | NGR69 | -0.052 |
| NGR37 | NGR69 | -0.039 |
| NGR38 | NGR69 | -0.076 |
| NGR39 | NGR69 | -0.052 |
| NGR40 | NGR69 | -0.024 |
| NGR41 | NGR69 | 0.027 |
| NGR42 | NGR69 | -0.045 |
| NGR43 | NGR69 | -0.033 |
| NGR44 | NGR69 | -0.088 |
| NGR45 | NGR69 | -0.033 |
| NGR46 | NGR69 | -0.091 |
| NGR47 | NGR69 | -0.030 |
| NGR48 | NGR69 | 0.032 |
| NGR49 | NGR69 | -0.033 |
| NGR50 | NGR69 | -0.076 |
| NGR51 | NGR69 | 0.032 |
| NGR52 | NGR69 | -0.024 |
| NGR53 | NGR69 | 0.040 |
| NGR54 | NGR69 | -0.050 |
| NGR55 | NGR69 | -0.057 |
| NGR56 | NGR69 | -0.029 |
| NGR57 | NGR69 | -0.033 |
| NGR58 | NGR69 | 0.021 |
| NGR59 | NGR69 | -0.070 |
| NGR60 | NGR69 | -0.043 |
| NGR61 | NGR69 | -0.034 |
| NGR62 | NGR69 | -0.052 |
| NGR63 | NGR69 | -0.020 |
| NGR64 | NGR69 | 0.063 |
| NGR65 | NGR69 | 0.021 |
| NGR66 | NGR69 | -0.011 |
| NGR67 | NGR69 | 0.051 |
| NGR68 | NGR69 | 0.003 |
| NGR1 | NGR70 | 0.003 |
| NGR2 | NGR70 | -0.033 |
| NGR3 | NGR70 | -0.046 |
| NGR4 | NGR70 | -0.082 |
| NGR5 | NGR70 | -0.054 |
| NGR6 | NGR70 | -0.110 |
| NGR7 | NGR70 | -0.052 |
| NGR8 | NGR70 | -0.164 |
| NGR9 | NGR70 | -0.090 |
| NGR10 | NGR70 | -0.015 |
| NGR11 | NGR70 | -0.070 |
| NGR12 | NGR70 | 0.045 |
| NGR13 | NGR70 | -0.052 |
| NGR14 | NGR70 | 0.051 |
| NGR15 | NGR70 | -0.070 |
| NGR16 | NGR70 | -0.070 |
| NGR17 | NGR70 | -0.064 |
| NGR18 | NGR70 | 0.041 |
| NGR19 | NGR70 | 0.009 |
| NGR20 | NGR70 | -0.082 |
| NGR21 | NGR70 | -0.052 |
| NGR22 | NGR70 | -0.121 |
| NGR23 | NGR70 | -0.111 |
| NGR24 | NGR70 | -0.082 |
| NGR25 | NGR70 | -0.100 |
| NGR26 | NGR70 | -0.079 |
| NGR27 | NGR70 | -0.052 |
| NGR28 | NGR70 | -0.100 |
| NGR29 | NGR70 | -0.043 |
| NGR30 | NGR70 | -0.052 |
| NGR31 | NGR70 | 0.048 |
| NGR32 | NGR70 | -0.048 |
| NGR33 | NGR70 | -0.061 |
| NGR34 | NGR70 | -0.052 |
| NGR35 | NGR70 | 0.027 |
| NGR36 | NGR70 | -0.052 |
| NGR37 | NGR70 | -0.039 |
| NGR38 | NGR70 | -0.076 |
| NGR39 | NGR70 | -0.052 |
| NGR40 | NGR70 | -0.024 |
| NGR41 | NGR70 | 0.027 |
| NGR42 | NGR70 | -0.045 |
| NGR43 | NGR70 | -0.033 |
| NGR44 | NGR70 | -0.088 |
| NGR45 | NGR70 | -0.033 |
| NGR46 | NGR70 | -0.091 |
| NGR47 | NGR70 | -0.085 |
| NGR48 | NGR70 | 0.032 |
| NGR49 | NGR70 | -0.033 |
| NGR50 | NGR70 | 0.215 |
| NGR51 | NGR70 | 0.322 |
| NGR52 | NGR70 | -0.024 |
| NGR53 | NGR70 | -0.015 |
| NGR54 | NGR70 | -0.073 |
| NGR55 | NGR70 | -0.057 |
| NGR56 | NGR70 | -0.052 |
| NGR57 | NGR70 | -0.033 |
| NGR58 | NGR70 | -0.033 |
| NGR59 | NGR70 | -0.070 |
| NGR60 | NGR70 | -0.043 |
| NGR61 | NGR70 | 0.052 |
| NGR62 | NGR70 | -0.052 |
| NGR63 | NGR70 | -0.052 |
| NGR64 | NGR70 | 0.008 |
| NGR65 | NGR70 | -0.033 |
| NGR66 | NGR70 | -0.033 |
| NGR67 | NGR70 | 0.432 |
| NGR68 | NGR70 | 0.166 |
| NGR69 | NGR70 | 0.614 |
| NGR1 | NGR71 | -0.051 |
| NGR2 | NGR71 | 0.109 |
| NGR3 | NGR71 | -0.121 |
| NGR4 | NGR71 | -0.020 |
| NGR5 | NGR71 | -0.069 |
| NGR6 | NGR71 | -0.093 |
| NGR7 | NGR71 | 0.018 |
| NGR8 | NGR71 | -0.146 |
| NGR9 | NGR71 | -0.074 |
| NGR10 | NGR71 | -0.053 |
| NGR11 | NGR71 | -0.068 |
| NGR12 | NGR71 | -0.033 |
| NGR13 | NGR71 | -0.053 |
| NGR14 | NGR71 | -0.067 |
| NGR15 | NGR71 | 0.000 |
| NGR16 | NGR71 | 0.082 |
| NGR17 | NGR71 | 0.064 |
| NGR18 | NGR71 | -0.059 |
| NGR19 | NGR71 | -0.038 |
| NGR20 | NGR71 | 0.114 |
| NGR21 | NGR71 | 0.022 |
| NGR22 | NGR71 | -0.024 |
| NGR23 | NGR71 | 0.032 |
| NGR24 | NGR71 | 0.087 |
| NGR25 | NGR71 | -0.012 |
| NGR26 | NGR71 | -0.046 |
| NGR27 | NGR71 | 0.000 |
| NGR28 | NGR71 | -0.031 |
| NGR29 | NGR71 | -0.006 |
| NGR30 | NGR71 | 0.017 |
| NGR31 | NGR71 | -0.090 |
| NGR32 | NGR71 | 0.055 |
| NGR33 | NGR71 | -0.070 |
| NGR34 | NGR71 | 0.002 |
| NGR35 | NGR71 | 0.058 |
| NGR36 | NGR71 | 0.018 |
| NGR37 | NGR71 | 0.016 |
| NGR38 | NGR71 | 0.018 |
| NGR39 | NGR71 | -0.045 |
| NGR40 | NGR71 | 0.007 |
| NGR41 | NGR71 | 0.066 |
| NGR42 | NGR71 | 0.085 |
| NGR43 | NGR71 | 0.016 |
| NGR44 | NGR71 | -0.036 |
| NGR45 | NGR71 | -0.007 |
| NGR46 | NGR71 | 0.061 |
| NGR47 | NGR71 | 0.014 |
| NGR48 | NGR71 | 0.048 |
| NGR49 | NGR71 | -0.003 |
| NGR50 | NGR71 | 0.083 |
| NGR51 | NGR71 | -0.051 |
| NGR52 | NGR71 | -0.036 |
| NGR53 | NGR71 | -0.010 |
| NGR54 | NGR71 | 0.107 |
| NGR55 | NGR71 | 0.152 |
| NGR56 | NGR71 | 0.003 |
| NGR57 | NGR71 | -0.027 |
| NGR58 | NGR71 | 0.083 |
| NGR59 | NGR71 | -0.045 |
| NGR60 | NGR71 | 0.026 |
| NGR61 | NGR71 | 0.038 |
| NGR62 | NGR71 | -0.027 |
| NGR63 | NGR71 | -0.026 |
| NGR64 | NGR71 | 0.043 |
| NGR65 | NGR71 | -0.021 |
| NGR66 | NGR71 | -0.075 |
| NGR67 | NGR71 | -0.101 |
| NGR68 | NGR71 | -0.035 |
| NGR69 | NGR71 | -0.094 |
| NGR70 | NGR71 | -0.094 |
| NGR1 | NGR72 | -0.027 |
| NGR2 | NGR72 | -0.023 |
| NGR3 | NGR72 | -0.121 |
| NGR4 | NGR72 | 0.068 |
| NGR5 | NGR72 | 0.014 |
| NGR6 | NGR72 | -0.110 |
| NGR7 | NGR72 | -0.023 |
| NGR8 | NGR72 | -0.146 |
| NGR9 | NGR72 | -0.086 |
| NGR10 | NGR72 | -0.004 |
| NGR11 | NGR72 | -0.064 |
| NGR12 | NGR72 | 0.050 |
| NGR13 | NGR72 | 0.072 |
| NGR14 | NGR72 | -0.044 |
| NGR15 | NGR72 | -0.041 |
| NGR16 | NGR72 | 0.026 |
| NGR17 | NGR72 | -0.006 |
| NGR18 | NGR72 | -0.020 |
| NGR19 | NGR72 | 0.000 |
| NGR20 | NGR72 | -0.024 |
| NGR21 | NGR72 | -0.050 |
| NGR22 | NGR72 | -0.077 |
| NGR23 | NGR72 | 0.023 |
| NGR24 | NGR72 | -0.074 |
| NGR25 | NGR72 | -0.040 |
| NGR26 | NGR72 | -0.068 |
| NGR27 | NGR72 | -0.023 |
| NGR28 | NGR72 | -0.058 |
| NGR29 | NGR72 | 0.020 |
| NGR30 | NGR72 | 0.066 |
| NGR31 | NGR72 | -0.039 |
| NGR32 | NGR72 | 0.020 |
| NGR33 | NGR72 | -0.047 |
| NGR34 | NGR72 | 0.023 |
| NGR35 | NGR72 | 0.111 |
| NGR36 | NGR72 | -0.023 |
| NGR37 | NGR72 | 0.027 |
| NGR38 | NGR72 | -0.034 |
| NGR39 | NGR72 | -0.032 |
| NGR40 | NGR72 | 0.029 |
| NGR41 | NGR72 | 0.031 |
| NGR42 | NGR72 | -0.007 |
| NGR43 | NGR72 | 0.029 |
| NGR44 | NGR72 | -0.077 |
| NGR45 | NGR72 | 0.007 |
| NGR46 | NGR72 | 0.034 |
| NGR47 | NGR72 | 0.023 |
| NGR48 | NGR72 | -0.002 |
| NGR49 | NGR72 | 0.020 |
| NGR50 | NGR72 | 0.011 |
| NGR51 | NGR72 | -0.012 |
| NGR52 | NGR72 | -0.013 |
| NGR53 | NGR72 | 0.084 |
| NGR54 | NGR72 | 0.043 |
| NGR55 | NGR72 | 0.008 |
| NGR56 | NGR72 | 0.115 |
| NGR57 | NGR72 | 0.011 |
| NGR58 | NGR72 | 0.023 |
| NGR59 | NGR72 | -0.016 |
| NGR60 | NGR72 | 0.020 |
| NGR61 | NGR72 | 0.090 |
| NGR62 | NGR72 | 0.027 |
| NGR63 | NGR72 | -0.023 |
| NGR64 | NGR72 | 0.059 |
| NGR65 | NGR72 | 0.088 |
| NGR66 | NGR72 | 0.034 |
| NGR67 | NGR72 | -0.013 |
| NGR68 | NGR72 | 0.013 |
| NGR69 | NGR72 | 0.000 |
| NGR70 | NGR72 | -0.045 |
| NGR71 | NGR72 | 0.035 |
| NGR1 | NGR73 | -0.033 |
| NGR2 | NGR73 | -0.015 |
| NGR3 | NGR73 | -0.121 |
| NGR4 | NGR73 | -0.057 |
| NGR5 | NGR73 | -0.050 |
| NGR6 | NGR73 | -0.093 |
| NGR7 | NGR73 | 0.075 |
| NGR8 | NGR73 | -0.146 |
| NGR9 | NGR73 | -0.079 |
| NGR10 | NGR73 | -0.023 |
| NGR11 | NGR73 | -0.038 |
| NGR12 | NGR73 | -0.057 |
| NGR13 | NGR73 | -0.023 |
| NGR14 | NGR73 | -0.037 |
| NGR15 | NGR73 | 0.056 |
| NGR16 | NGR73 | 0.030 |
| NGR17 | NGR73 | 0.091 |
| NGR18 | NGR73 | -0.028 |
| NGR19 | NGR73 | -0.008 |
| NGR20 | NGR73 | 0.028 |
| NGR21 | NGR73 | -0.034 |
| NGR22 | NGR73 | -0.001 |
| NGR23 | NGR73 | -0.018 |
| NGR24 | NGR73 | -0.071 |
| NGR25 | NGR73 | -0.031 |
| NGR26 | NGR73 | -0.017 |
| NGR27 | NGR73 | 0.029 |
| NGR28 | NGR73 | -0.057 |
| NGR29 | NGR73 | -0.019 |
| NGR30 | NGR73 | 0.003 |
| NGR31 | NGR73 | -0.060 |
| NGR32 | NGR73 | 0.028 |
| NGR33 | NGR73 | -0.053 |
| NGR34 | NGR73 | -0.023 |
| NGR35 | NGR73 | -0.012 |
| NGR36 | NGR73 | 0.075 |
| NGR37 | NGR73 | -0.009 |
| NGR38 | NGR73 | 0.063 |
| NGR39 | NGR73 | -0.015 |
| NGR40 | NGR73 | -0.006 |
| NGR41 | NGR73 | -0.004 |
| NGR42 | NGR73 | -0.021 |
| NGR43 | NGR73 | 0.029 |
| NGR44 | NGR73 | 0.020 |
| NGR45 | NGR73 | -0.019 |
| NGR46 | NGR73 | -0.007 |
| NGR47 | NGR73 | -0.044 |
| NGR48 | NGR73 | -0.019 |
| NGR49 | NGR73 | -0.015 |
| NGR50 | NGR73 | 0.082 |
| NGR51 | NGR73 | -0.064 |
| NGR52 | NGR73 | -0.006 |
| NGR53 | NGR73 | -0.023 |
| NGR54 | NGR73 | -0.066 |
| NGR55 | NGR73 | -0.027 |
| NGR56 | NGR73 | -0.010 |
| NGR57 | NGR73 | 0.003 |
| NGR58 | NGR73 | -0.041 |
| NGR59 | NGR73 | 0.011 |
| NGR60 | NGR73 | 0.014 |
| NGR61 | NGR73 | 0.011 |
| NGR62 | NGR73 | 0.003 |
| NGR63 | NGR73 | 0.003 |
| NGR64 | NGR73 | -0.012 |
| NGR65 | NGR73 | -0.034 |
| NGR66 | NGR73 | -0.045 |
| NGR67 | NGR73 | -0.077 |
| NGR68 | NGR73 | -0.006 |
| NGR69 | NGR73 | -0.064 |
| NGR70 | NGR73 | -0.064 |
| NGR71 | NGR73 | 0.064 |
| NGR72 | NGR73 | -0.006 |
