# Supplementary material for: Predictive genetic plan for a captive population of the Chinese goral (Naemorhedus griseus) and prescriptive action for ex situ and in situ conservation management in Thailand
Source: PLoS One. 2020 Jun 4;15(6):e0234064. doi: 10.1371/journal.pone.0234064 (PMC7272075; doi:10.1371/journal.pone.0234064)
Supplement: S8 Table — Detailed information for all N. griseus individuals is presented in S1 Table. (DOCX) [file pone.0234064.s008.docx]

**Table S8.** Pairwise inbreeding coefficients (*F*_IS_) for all 73 *Naemorhedus griseus* individuals. Detailed information for all *N. griseus* individuals is presented in Table S2.

| Individual | FIS | Individual | *F*_IS_ |
| --- | --- | --- | --- |
| NGR1 | -0.0721 | NGR38 | -0.0454 |
| NGR2 | 0.0682 | NGR39 | -0.0635 |
| NGR3 | 0.6233 | NGR40 | 0.0135 |
| NGR4 | -0.0562 | NGR41 | 0.003 |
| NGR5 | 0.0537 | NGR42 | -0.0521 |
| NGR6 | 0.0258 | NGR43 | -0.001 |
| NGR7 | -0.042 | NGR44 | 0.0189 |
| NGR8 | 1.7174 | NGR45 | 0.002 |
| NGR9 | 0.4279 | NGR46 | -0.0958 |
| NGR10 | 0.0084 | NGR47 | -0.033 |
| NGR11 | 0.5548 | NGR48 | 0.073 |
| NGR12 | 0.0743 | NGR49 | 0.0711 |
| NGR13 | 0.0258 | NGR50 | -0.0596 |
| NGR14 | 0.1061 | NGR51 | 0.0333 |
| NGR15 | -0.0177 | NGR52 | 0.0299 |
| NGR16 | -0.0869 | NGR53 | 0.0077 |
| NGR17 | -0.0567 | NGR54 | -0.0451 |
| NGR18 | -0.0408 | NGR55 | 0.0742 |
| NGR19 | -0.0503 | NGR56 | 0.0077 |
| NGR20 | -0.0911 | NGR57 | 0.0064 |
| NGR21 | -0.0067 | NGR58 | 0.0153 |
| NGR22 | 0.0343 | NGR59 | -0.0024 |
| NGR23 | 0.0311 | NGR60 | -0.0076 |
| NGR24 | -0.1082 | NGR61 | -0.0549 |
| NGR25 | 0.0043 | NGR62 | 0.0558 |
| NGR26 | -0.081 | NGR63 | -0.035 |
| NGR27 | -0.0418 | NGR64 | 0.0267 |
| NGR28 | 0.0512 | NGR65 | -0.0701 |
| NGR29 | -0.0413 | NGR66 | -0.0481 |
| NGR30 | -0.0235 | NGR67 | 0.089 |
| NGR31 | -0.0897 | NGR68 | -0.0982 |
| NGR32 | 0.0333 | NGR69 | 0.0916 |
| NGR33 | 0.0648 | NGR70 | 0.056 |
| NGR34 | 0.0095 | NGR71 | 0.0525 |
| NGR35 | 0.0175 | NGR72 | -0.0437 |
| NGR36 | -0.0572 | NGR73 | -0.0325 |
| NGR37 | 0.0649 |  |  |
