# Supplementary material for: Predictive genetic plan for a captive population of the Chinese goral (Naemorhedus griseus) and prescriptive action for ex situ and in situ conservation management in Thailand
Source: PLoS One. 2020 Jun 4;15(6):e0234064. doi: 10.1371/journal.pone.0234064 (PMC7272075; doi:10.1371/journal.pone.0234064)
Supplement: S9 Table — Detailed information for all N. griseus individuals is presented in S1 Table. (DOCX) [file pone.0234064.s009.docx]

**Table S9**. Parentage analysis of 73 *Naemorhedus griseus* individuals. Detailed information for all *N. griseus* individuals is presented in Table S2.

| FullSibship  Index | Prob  (Inc.) | Prob  (Exc.) | Member1 | Member2 | Member3 | Member4 | Member5 | Member6 |
| --- | --- | --- | --- | --- | --- | --- | --- | --- |
| 1 | 0.3996 | 0.0776 | NGR1 | NGR38 |  |  |  |  |
| 2 | 0.5542 | 0.5047 | NGR2 | NGR37 | NGR43 | NGR55 |  |  |
| 3 | 1 | 1 | NGR3 |  |  |  |  |  |
| 4 | 0.9337 | 0.9337 | NGR4 | NGR54 |  |  |  |  |
| 5 | 1 | 1 | NGR5 |  |  |  |  |  |
| 6 | 0.9671 | 0.9671 | NGR6 | NGR8 |  |  |  |  |
| 7 | 0.8762 | 0.8227 | NGR7 | NGR17 | NGR27 | NGR36 | NGR73 |  |
| 8 | 0.9899 | 0.825 | NGR9 | NGR28 |  |  |  |  |
| 9 | 1 | 1 | NGR10 |  |  |  |  |  |
| 10 | 0.8457 | 0.8457 | NGR11 | NGR45 |  |  |  |  |
| 11 | 0.9008 | 0.4941 | NGR12 | NGR35 | NGR64 |  |  |  |
| 12 | 0.7822 | 0.7746 | NGR13 | NGR53 | NGR56 | NGR57 | NGR62 | NGR65 |
| 13 | 0.931 | 0.931 | NGR14 | NGR52 |  |  |  |  |
| 14 | 0.9505 | 0.9505 | NGR15 | NGR59 |  |  |  |  |
| 15 | 0.7856 | 0.5327 | NGR16 | NGR58 |  |  |  |  |
| 16 | 1 | 0.3223 | NGR18 |  |  |  |  |  |
| 17 | 1 | 1 | NGR19 |  |  |  |  |  |
| 18 | 1 | 0.1715 | NGR20 |  |  |  |  |  |
| 19 | 1 | 0.3223 | NGR21 |  |  |  |  |  |
| 20 | 0.9789 | 0.4676 | NGR22 | NGR44 |  |  |  |  |
| 21 | 1 | 0.3722 | NGR23 |  |  |  |  |  |
| 22 | 1 | 0.3035 | NGR24 |  |  |  |  |  |
| 23 | 1 | 0.2022 | NGR25 |  |  |  |  |  |
| 24 | 0.6872 | 0.6872 | NGR26 | NGR33 |  |  |  |  |
| 25 | 1 | 0.208 | NGR29 |  |  |  |  |  |
| 26 | 0.4902 | 0.4902 | NGR30 | NGR68 |  |  |  |  |
| 27 | 0.9227 | 0.8825 | NGR31 | NGR66 |  |  |  |  |
| 28 | 1 | 0.9061 | NGR32 |  |  |  |  |  |
| 29 | 0.8966 | 0.4767 | NGR34 | NGR47 |  |  |  |  |
| 30 | 1 | 0.3747 | NGR39 |  |  |  |  |  |
| 31 | 0.8508 | 0.2735 | NGR40 | NGR49 |  |  |  |  |
| 32 | 1 | 0.1933 | NGR41 |  |  |  |  |  |
| 33 | 1 | 1 | NGR42 |  |  |  |  |  |
| 34 | 1 | 1 | NGR46 |  |  |  |  |  |
| 35 | 1 | 0.4517 | NGR48 |  |  |  |  |  |
| 36 | 0.9138 | 0.9138 | NGR50 | NGR61 |  |  |  |  |
| 37 | 1 | 1 | NGR51 |  |  |  |  |  |
| 38 | 1 | 0.2105 | NGR60 |  |  |  |  |  |
| 39 | 1 | 0.3838 | NGR63 |  |  |  |  |  |
| 40 | 0.8735 | 0.8735 | NGR67 | NGR69 | NGR70 |  |  |  |
| 41 | 1 | 1 | NGR71 |  |  |  |  |  |
| 42 | 1 | 0.1224 | NGR72 |  |  |  |  |  |
