# Supplementary material for: Predictive genetic plan for a captive population of the Chinese goral (Naemorhedus griseus) and prescriptive action for ex situ and in situ conservation management in Thailand
Source: PLoS One. 2020 Jun 4;15(6):e0234064. doi: 10.1371/journal.pone.0234064 (PMC7272075; doi:10.1371/journal.pone.0234064)
Supplement: S10 Table — Detailed information for all N. griseus individuals is presented in S1 Table. (DOCX) [file pone.0234064.s010.docx]

**Table S10.** Probability of identity estimated using Gimlet version 1.3.3 (Valière, 2002) of *Naemorhedus griseus* individuals based on 11 microsatellite loci. Detailed information for all *N. griseus* individuals is presented in Table S1.

| Locus | Unbias/loc. | Prod(unbias) |
| --- | --- | --- |
| SY434F | 4.50E-01 | 4.50E-01 |
| SY14F | 2.22E-01 | 9.97E-02 |
| SY259F | 7.96E-01 | 7.93E-02 |
| SY12BF | 6.02E-02 | 4.77E-03 |
| SY93F | 1.58E-01 | 7.56E-04 |
| SY129F | 4.49E-01 | 3.39E-04 |
| SY76F | 4.44E-01 | 1.51E-04 |
| SY449F | 5.51E-01 | 8.30E-05 |
| SY128F | 7.45E-02 | 6.19E-06 |
| SY84BF | 4.32E-01 | 2.67E-06 |
| SY84F | 2.59E-01 | 6.91E-07 |
| Mean | 3.54E-01 | 5.77E-02 |
| S.D. | 0.223 | 0.135 |
